# A missense mutation in *Kcnc3* causes hippocampal learning deficits in mice

**DOI:** 10.1073/pnas.2204901119

**Published:** 2022-07-26

**Authors:** Pin Xu, Kazuhiro Shimomura, Changhoon Lee, Xiaofei Gao, Eleanor H. Simpson, Guocun Huang, Chryshanthi M. Joseph, Vivek Kumar, Woo-Ping Ge, Karen S. Pawlowski, Mitchell D. Frye, Saïd Kourrich, Eric R. Kandel, Joseph S. Takahashi

**Affiliations:** ^a^Department of Neuroscience, University of Texas Southwestern Medical Center, Dallas, TX 75390;; ^b^Department of Neurobiology, Northwestern University, Evanston, IL 60208;; ^c^Children's Medical Center Research Institute, University of Texas Southwestern Medical Center, Dallas, TX 75390;; ^d^Department of Psychiatry, Columbia University, New York, NY 10032;; ^e^Research Foundation for Mental Hygiene, New York State Psychiatric Institute, New York, NY 10032;; ^f^Department of Pediatrics, University of Texas Southwestern Medical Center, Dallas, TX 75390-9152;; ^g^Department of Psychiatry, University of Texas Southwestern Medical Center, Dallas, TX 75390;; ^h^Department of Neuroscience, Columbia University, New York, NY 10032;; ^i^Kavli Institute of Brain Science, Columbia University, New York, NY 10032;; ^j^Mortimer B. Zuckerman Mind Brain Behavior Institute, Columbia University, New York, NY 10032;; ^k^HHMI, Columbia University, New York, NY 10032;; ^l^HHMI, University of Texas Southwestern Medical Center, Dallas, TX 75390

**Keywords:** learning and memory, potassium channels, behavioral screen, ENU mutagenesis, hippocampus

## Abstract

Although a wide variety of genetic tools has been developed to study learning and memory, the molecular basis of memory encoding remains incompletely understood. Here, we undertook an unbiased approach to identify novel genes critical for memory encoding. From a large-scale, in vivo mutagenesis screen using contextual fear conditioning, we isolated in mice a mutant, named *Clueless*, with spatial learning deficits. A causative missense mutation (G434V) was found in the voltage-gated potassium channel, subfamily C member 3 (*Kcnc3)* gene in a region that encodes a transmembrane voltage sensor. Generation of a Kcnc3^G434V^ CRISPR mutant mouse confirmed this mutation as the cause of the learning defects. While G434V had no effect on transcription, translation, or trafficking of the channel, electrophysiological analysis of the G434V mutant channel revealed a complete loss of voltage-gated conductance, a broadening of the action potential, and decreased neuronal firing. Together, our findings have revealed a role for *Kcnc3* in learning and memory.

## Significance

Using an unbiased genetic screen, we uncovered a novel missense mutation in the voltage-gated potassium channel, subfamily C member 3 gene (*Kcnc3)* that decreases the activity of hippocampal neurons and causes defects in learning and memory in a fear-conditioning task. These findings provide evidence that *Kcnc3* is important for hippocampal encoding of memories and contribute significantly to our understanding of potassium currents in the hippocampus and their role in learning.

Learning is the physiological process of acquiring knowledge about the world, and memory is the storage of this knowledge over time (1, 2). These are complex processes that involve many genes, cell types, and brain circuits (1, 3–9). The development of optogenetic and chemogenetic approaches has greatly expanded our knowledge of the circuitry underlying memory processes (7, 10–14), and transcriptomics has also revealed molecular changes that correlate with learning and memory storage (15–19). Despite this body of work, our understanding of the molecular basis of learning and memory remains incomplete.

In mammals, reverse genetics (from gene to phenotype) has been widely used to test candidate genes for their roles in synaptic transmission, synaptic plasticity, and signal transduction (6, 20–25). In contrast, forward genetic approaches (from phenotype to gene) for unbiased gene discovery have rarely been attempted in the learning and memory field (26–28), although this strategy has been successfully used for studying fear behaviors in mice (29), as well as learning and memory in *Drosophila* (30–33). Here, we report the findings of an unbiased forward genetic screen to identify regulators of learning and memory in mice. We identify and validate a missense mutation in the voltage-gated potassium channel, subfamily C member 3 *Kcnc3* gene that is associated with learning deficits and show that it alters the firing properties of hippocampal neurons and the expression of genes in the hippocampus after fear conditioning.

## Results

### A Forward Genetics Behavioral Screen Reveals Mice with Learning Deficits.

**Fig. 1.**
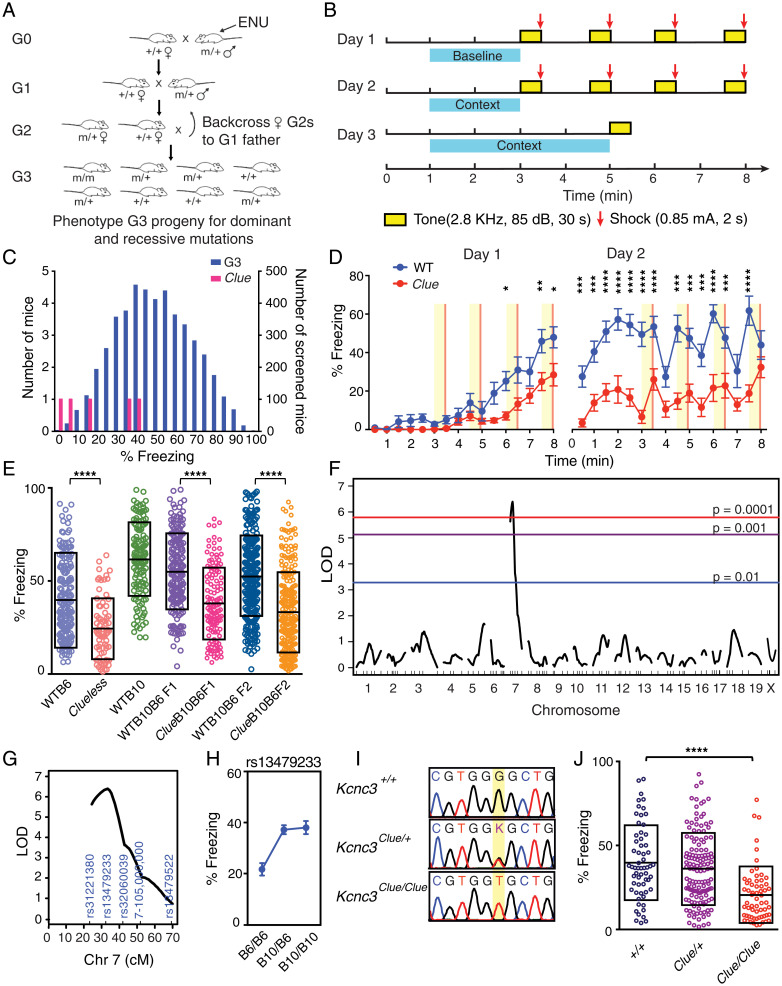
Forward genetic screening using contextual fear conditioning reveals a low freezing mutant. (*A*) Breeding schematic for behavioral screening. See details in *Materials and Methods*. (*B*) Design of the fear-conditioning screening protocol. Each block represents 30 s. (*C*) Phenotypic distribution of freezing scores of G3 (*n* = 4,691; blue bars) and *Clueless* (*n* = 5; magenta bars) mice. (*D*) Percent freezing time in *Clueless* and WT B6J mice during training (males, *n* = 12 for both groups). Data are reported as mean ± SEM. ****P < 0.0001, ***P < 0.001, **P < 0.01, *P < 0.05. Two-way repeated measures ANOVA results for the following: genotype × time interaction, *F*_31,341_ = 5.556, *P* < 0.001; genotype effect, *F*_1,11_ = 41.34, *P* < 0.0001; time effect, *F*_31,341_ = 5.556, *P* < 0.001, adjusted with Sidak's post hoc test. (*E*) Percent freezing time in mice of different genotypes. *Clueless* mutants showed decreased freezing behavior when maintained on either C57BL/6J or a mixed B10B6 background. WTB6, *n* = 178; *Clueless*, *n* = 74; WTB10, *n* = 128; WTB10B6F1, *n* = 174; WTB10B6F2, *n* = 264; *Clue*B10B6F1, *n* = 131; and *Clue*B10B6F2, *n* = 259. Data are reported as mean ± SD. ****P < 0.0001, Kolmogorov-Smirnov test. (*F*) QTL analysis of *Clueless*. The significance thresholds were calculated with 10,000 permutation tests. (*G*) The LOD score of the significant QTL on chromosome (Chr) 7 peaks near rs13479233. (*H*) Genotype effect plot of rs13479233. Data are reported as mean ± SEM. (*I*) Genotypes of *Clueless* mutants from Sanger sequencing. (*J*) Percent freezing time in *Kcnc3*^+/+^ (*n* = 61), *Kcnc3^Clue^*^/+^ (*n* = 136), and *Kcnc3^Clue/Clue^* (*n* = 62) mice. Data are reported as mean ± SD. *F*_2,256_ = 17.33. ****P < 0.0001, one-way ANOVA, adjusted with Tukey’s post hoc test. *Clue*, *Clueless*.

Male C57BL/6J (B6J) mice (G0) were treated with three doses of *N*-ethyl-*N*-nitrosourea (ENU) (once per week) and were mated with wild-type (WT) B6J females to generate progeny (G1) that carry random point mutations in the genome. We then bred individual G1 males with WT B6J females to produce female progeny (G2) that were subsequently backcrossed to their G1 fathers to produce G3 mice for behavioral screening (breeding scheme in Fig. 1*A*) (34). More than 20,000 G3 mice were screened using a contextual fear-conditioning testing (FCT) protocol (Fig. 1*B*) (35). Our screening strategy utilized context-dependent and cued fear conditioning, which are robust behaviors that can be acquired in a single training session. Comparison of contextual and cued FCT responses can then be used to bias the screen toward either hippocampal- or amygdala-associated components.

Data from a cohort of 4,691 screened G3 mice are shown in Fig. 1*C*. Pedigrees that showed either abnormally high or low freezing behavior 24 h after training (≥1 SD from the mean of all mice tested) were selected for further analyses. Among these, one pedigree had five mice with extremely low freezing scores. These mice also showed reduced freezing behavior during training, suggesting a defect in learning (Fig. 1*D*). We therefore named the mutant mice *Clueless.* The *Clueless* line was maintained by intercrossing littermates with the lowest freezing behavior. Learning and memory deficits persisted in all subsequent generations (Fig. 1*E*).

### Defects in Learning and Memory in *Clueless* Mutants Map to a Mutation in *Kcnc3*.

To map the location of *Clueless* in the genome, we mated *Clueless* males (in B6J background) with WT C57BL/10J (B10J) females to produce F2 mapping mice (*Clue*B10B6F2) (Fig. 1*E* and *SI Appendix*, Fig. S1) for quantitative trait locus (QTL) analysis (36) using a single nucleotide polymorphism (SNP) panel between B6J and B10J (*SI Appendix*, Fig. S1 and Dataset S1). A genome scan revealed one significant QTL on chromosome 7 (log of odds [LOD] = 6.4) (Fig. 1*F*) that contributed to 10.8% of the total observed phenotypic variance (genetic, environment, and error) and 95.8% of the genetic variance (*SI Appendix*, Fig. S1). The LOD support interval was estimated to be ∼16 cM, between rs31221380 and rs32060039, with rs13479233 being the closest SNP to the peak of the QTL (Fig. 1*G*). Furthermore, the effect plot of rs13479233 showed that mice with a B6J genotype had low freezing behavior, whereas those with a B10J genotype had high freezing behavior (Fig. 1*H*). Although WT B10J mice had higher freezing scores compared with WT B6J mice, a genome scan failed to reveal any significant QTLs in this control cross for genetic background (*SI Appendix*, Fig. S2), suggesting that there were no background QTLs from B10J masking the *Clueless* trait.

To identify the causative mutation, we conducted whole-exome sequencing and found a single nonsynonymous-coding SNP within the LOD support interval. The SNP (G > T, chr7:44,595,588, mm10) was located in the second exon of *Kcnc3*, resulting in a missense mutation from glycine to valine (KCNC3 G434V) (Dataset S2). The G > T transition was further confirmed by Sanger sequencing (Fig. 1*I*). We then used allele-specific PCR to genotype all *Clue*B10B6F2 mice and found that homozygous *Clueless* mutants of both sexes showed 46% less freezing behavior (1 SD) compared with their WT littermates (Fig. 1*J* and *SI Appendix*, Fig. S3). Together, these data suggested that the G > T transition was likely the causative mutation in *Clueless* mice.

### *Clueless* Mutants Have Defects Specifically in Spatial Learning Tasks.

**Fig. 2.**
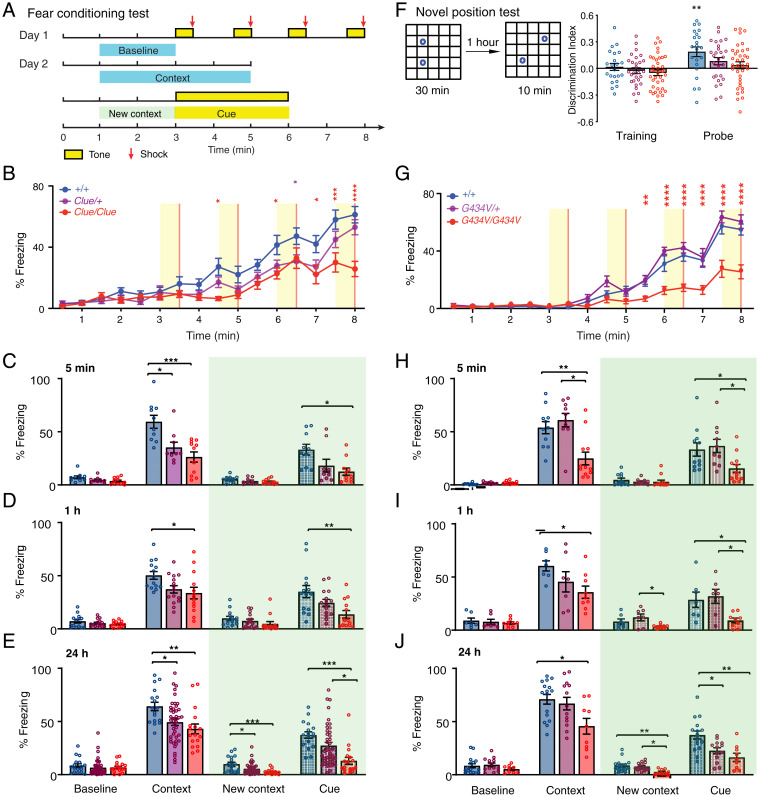
Spatial-learning deficits in *Clueless* (*Clue*) mice are specific and due to the G434V mutation. (*A*) Schematic of the fear-conditioning test. (*B*) Percent freezing time during training, ^+/+^ (*n* = 18), *Clue*/+ (*n* = 20), *Clue/Clue* (*n* = 16). Data are reported as mean ± SEM. ****P < 0.0001, ***P < 0.001, **P < 0.01, *P < 0.05. Two-way repeated measures ANOVA results: genotype × time interaction, *F*_30,765_ = 3.305, ****P < 0.0001; time effect, *F*_15,765_ = 65.63, ****P < 0.0001; genotype effect, *F*_2,51_ = 6.769, **P = 0.0025, adjusted with Tukey’s post hoc test. (*C*) Percent freezing time tested 5 min after training: ^+/+^, *n* = 10; *Clue*/+, *n* = 9; *Clue/Clue*, *n* = 11. ***P < 0.001, *P < 0.05. Results of one-way ANOVA adjusted with Tukey’s post hoc test: contextual % freezing, *F*_2,27_ = 10.66, *P* = 0.0004; cued % freezing, *F*_2,27_ = 5.086, *P* = 0.0133; baseline % freezing, *F*_2,27_ = 2.974, *P* = 0.0680; changed context baseline % freezing, *F*_2,27_ = 2.103, *P* = 0.1416. (*D*) Percent freezing time tested 1 h after training: ^+/+^, *n* = 14; *Clue*/+, *n* = 15; *Clue/Clue*, *n* = 13. **P < 0.01, *P < 0.05, one-way ANOVA adjusted with Tukey’s post hoc test. Contextual % freezing, *F*_2,39_ = 4.313, *P* = 0.0203; cued % freezing, *F*_2,39_ = 5.81, *P* = 0.0062; baseline % freezing, *F*_2,39_ = 1.257, *P* = 0.2957; changed context baseline % freezing, *F*_2,39_ = 1.695, *P* = 0.1968. (*E*) Percent freezing time tested 24 h after training: ^+/+^, *n* = 17; *Clue*/+, *n* = 43; *Clue/Clue*, *n* = 17. ***P < 0.001, **P < 0.01, *P < 0.05, one-way ANOVA adjusted with Tukey’s post hoc test. Contextual % freezing, *F*_2,74_ = 5.803, *P* = 0.0046; cued % freezing, *F*_2,74_ = 8.779, *P* = 0.0004; baseline % freezing, *F*_2,74_ = 0.5131, *P* = 0.6008; changed context baseline % freezing, *F*_2,74_ = 8.192, *P* = 0.0006. (*F*) Schematic for a novel position test. Mice were trained in the arena with two identical objects for 30 min. ^+/+^, *n* = 22; *Clue*/+, *n* = 28; *Clue/Clue*, *n* = 36. One-sample *t* test, two tailed, ^+/+^, *P* = 0.6617; *Clue*/+, *P* = 0.4473; *Clue/Clue*, *P* = 0.147. One hour later, one object was moved to a new location and the exploratory behavior to the objects was analyzed for 10 min. One-sample *t* test, two tailed, ^+/+^, **P = 0.0025; *Clue*/+, *P* = 0.054; *Clue/Clue*, *P* = 0.3706. (*G–J*) Data from CRISPR-Cas9 engineered *Kcnc3_*mutant line *L53*; (*G*) percentage of freezing time during training. ^+/+^, *n* = 14; *Kcnc3^G434V^*^/+^, *n* = 13; *Kcnc3^G434V/G434V^*, *n* = 14. Two-way repeated measures ANOVA results: genotype × time interaction, *F*_30,570_ = 7.901, ****P < 0.0001; time effect, *F*_15,570_ = 120.3, ****P < 0.0001; genotype effect, *F*_2,38_ = 27.01, ****P < 0.0001, adjusted with Tukey’s post hoc test. (*H*) Percentage of freezing time 5 min after training; ^+/+^, *n* = 11; *Kcnc3^G434V^*^/+^, *n* = 10; *Kcnc3^G434V/G434V^*, *n* = 12. ***P < 0.0001, * *P* < 0.05. Results of one-way ANOVA, adjusted with Tukey’s post hoc test: contextual % freezing, *F*_2,30_ = 10.44, ***P = 0.0004; cued % freezing, *F*_2,30_ = 5.078, *P = 0.0126; baseline % freezing, *F*_2,30_ = 3.14, *P* = 0.0578; changed context baseline % freezing, *F*_2,30_ = 0.5018, *P* = 0.6104. (*I*) Percent freezing time tested 1 h after training; ^+/+^, *n* = 7; *Kcnc3^G434V^*^/+^, *n* = 7; *Kcnc3^G434V/G434V^*, *n* = 9. *P < 0.05. Results of one-way ANOVA, adjusted with Tukey’s post hoc test: contextual % freezing, *F*_2,20_ = 3.547, *P = 0.0480; cued % freezing, *F*_2,20_ = 5.522, *P = 0.0123; baseline % freezing, *F*_2,20_ = 0.3523, *P* = 0.7073; changed context baseline % freezing, *F*_2,20_ = 4.009, *P = 0.0343. (*J*) Percent freezing time tested 24 h after training; ^+/+^, *n* = 16; *Kcnc3^G434V^*^/+^, *n* = 13; *Kcnc3^G434V/G434V^*, *n* = 9. *P < 0.05. Results of one-way ANOVA, adjusted with Tukey’s post hoc test: contextual % freezing, *F*_2,35_ = 4.816, *P = 0.0142; cued % freezing, *F*_2,35_ = 8.823, ***P = 0.0008; baseline % freezing, *F*_2,35_ = 1.085, *P* = 0.3491; changed context baseline % freezing, *F*_2,35_ = 5.318, **P = 0.0096. Data are reported as mean ± SEM.

To further analyze the learning deficit in *Clueless* mutants, we used an extended FCT protocol (Fig. 2*A*) to examine contextual and cued memory acquisition or learning (5 min after training), short-term memory (1 h after training), as well as long-term memory (24 h after training). As expected, *Clueless* mutants showed reduced freezing behavior during training (Fig. 2 *B* and *C*) as well as defects in both short-term memory (Fig. 2*D*) and long-term memory (Fig. 2*E*). The learning deficit was also present in female mice as well as in older male mice (*SI Appendix*, Fig. S4).

Since the expression of fear conditioning behavior depends on the integration of multiple sensory systems, we measured sensory parameters, such as foot-shock sensitivity, odor habituation, and hearing, but found no differences between genotypes (*SI Appendix*, Fig. S5 *A–C*). As emotional status can impact behavior in learning tasks (37), we also verified that *Clueless* mice did not have increased anxiety-like behaviors in open field tests (*SI Appendix*, Fig. S5 *D–H*). However, when compared with WT littermates, homozygous *Clueless* mice weighed slightly less, showed less locomotor habituation, and had an increase in total distance traveled in the open field (*SI Appendix*, Fig. S5 *I–M*). *Clueless* mice also exhibited mild tremor, although we did not observe any evidence of gross motor defects.

To overcome the potential influence of these phenotypes on the scoring of freezing, we performed two additional learning and memory tests. In the Barnes circular maze, a test for spatial memory, *Clueless* mutants learned significantly slower than their littermate controls (*SI Appendix*, Fig. S5*N*), but both genotypes eventually located the escape hole, and memory retention was comparable between genotypes 7 d after training (*SI Appendix*, Fig. S5*O*). Since both fear conditioning and Barnes maze tests rely on the use of negative reinforcers, we also employed a novel position test that is driven by the innate curiosity of the mouse (38). We found that WT and *Clueless* mice spent similar amounts of time exploring two identical objects during the training session. However, 1 h after training, only WT mice showed a preference for the object placed at a new location (Fig. 2*F*). Along with our fear-conditioning results, these findings indicate that *Clueless* mice have a primary defect in spatial learning.

Finally, to confirm that the G434V variant was the causative allele, we also generated a *Kcnc3^G434V^* mouse with CRISPR/Cas9 genome editing. Using the same extended FCT protocol shown in Fig. 2*A*, we found that *Kcnc3^G434V^* mice derived from two independent founder lines (L50 and L53) exhibited similar deficits in fear conditioning and novel position tests as *Clueless* (Fig. 2 *G–J* and *SI Appendix*, Fig. S6), confirming that the G > T (chr7:44,595,588) transition is the causative mutation in *Clueless* mice.

### *Clueless* Mutants Have Altered Gene Expression in Response to Fear Conditioning.

In order to investigate the molecular basis for the altered fear-conditioning response, a separate cohort of mice (both WT and *Clueless*) underwent contextual FCT, and the dorsal and ventral parts of the dentate gyrus (DG) and cornu ammonis (CA) of the hippocampus were dissected for stranded messenger RNA sequencing (mRNA-seq). Genome-wide gene expression analysis showed clear expression differences across the four parts of the hippocampus of WT mice, with and without fear conditioning (*SI Appendix*, Fig. S7*A*). Interestingly, *Kcnc3* expression varied across the hippocampal region. *Kcnc3* was more highly expressed in the dorsal hippocampus than the ventral hippocampus (*SI Appendix*, Fig. S7*B*) and in the DG than the CA of WT mice. Importantly, neither *Kcnc3* expression nor the expression of other K^+^ channels differed between WT and *Clueless* hippocampi (Dataset S3).

In response to fear conditioning, there were fewer immediate early genes that were differentially expressed in *Clueless* mice as compared with WT mice (*SI Appendix*, Fig. S7*C*). This was particularly apparent in the dorsal DG, where *Kcnc3* expression was the highest. DG granule cells play an essential role in spatial learning and memory (12, 39), thus altered KCNC3 function in this region could impact the molecular response to fear conditioning. *Npas4* is a neuronal, activity-dependent, immediate early gene and induces the expression of learning- and memory-regulated genes in the hippocampus (40). *Npas4* knockdown has also been shown to inhibit fear memory (41). Interestingly, there were also fewer *Npas4* target genes in *Clueless* mice that changed expression after FCT training as compared with WT mice (*SI Appendix*, Fig. S7*C*), suggesting that the *Kcnc3* mutation causes learning deficits, at least in part by reducing the activity of *Npas4* in response to fear conditioning.

### The *Clueless* Mutation, *Kcnc3^G434V^*, Impairs Potassium Conductance In Vitro.

**Fig. 3.**
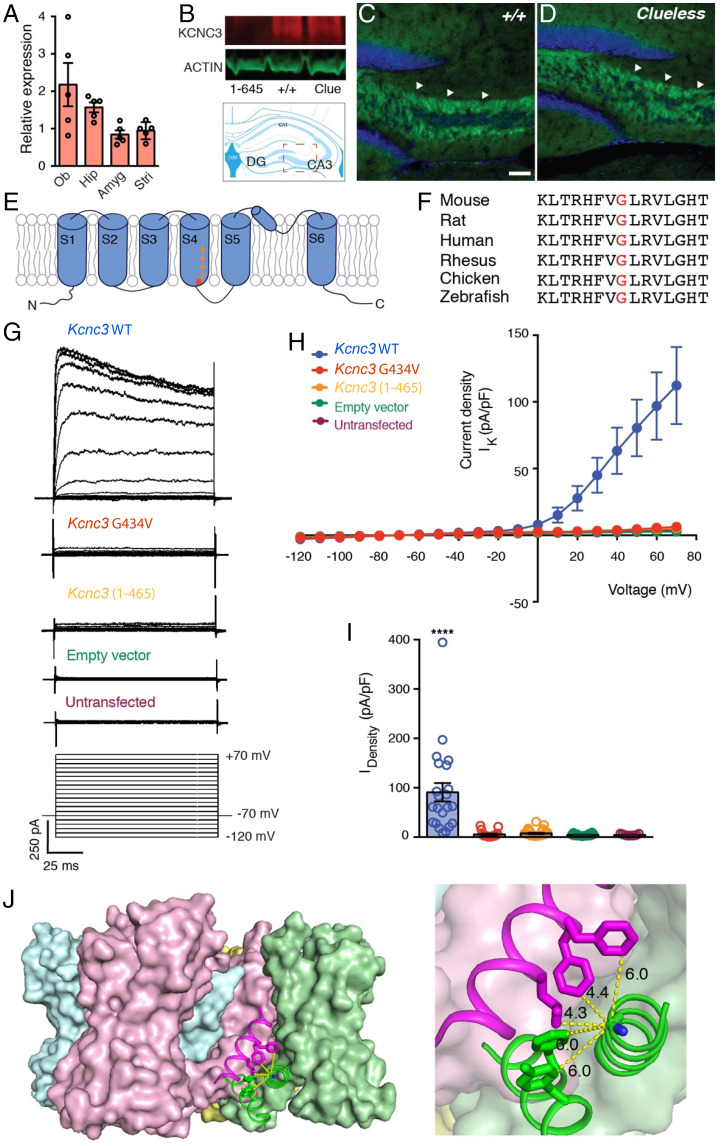
Functional characterization of KCNC3^G434V^ protein. (*A*) *Kcnc3* mRNA levels in olfactory bulb (Ob), hippocampus (Hip), amygdala (Amyg), and striatum (Stri) of 8-wk-old WT B6J mice (*n* = 5). (*B*) Western blot analysis of KCNC3 protein (red; ∼110 kDa) in hippocampus of *Kcnc3^(1-465)^*, *WT*, and *Clueless* (*Clue*) mutants. β-actin (shown in green; ∼45 kDa) was used as loading control. (*C* and *D*), Immunohistochemical analysis of KCNC3 (green) in hippocampus (arrow head, MFs) of both (*C*) WT and (*D*) *Clueless* mutants. Blue, nuclei staining by TOPRO3. Scale bars, 50 µm. (*E*) Schematic of α subunit of KCNC3 protein domains in lipid bilayers (G434; shown in red). (*F*) Alignment of partial KCNC3 protein sequences containing corresponding G434 (red) in multiple species. (*G*) Representative current traces of whole-cell voltage clamp recording of CHO cells transfected with either *Kcnc3^WT^*, *Kcnc3^G434V^*, *Kcnc3^(1-465)^*, or empty vectors. Cells were held at −70 mV and depolarized from −120 mV to 70 mV with 10-mV increments. (*H*) Current–voltage relationship plot of *Kcnc3^WT^* (blue; *n* = 17), *Kcnc3*^G434V^ (red; *n* = 13), *Kcnc3*^(1-465)^ (orange; *n* = 19), empty vector (green; *n* = 17) transfected CHO and untransfected CHO cells (purple; *n* = 9). (*I*) Current densities: *Kcnc3*, 90.90 ± 18.60 pA/pF; *Kcnc3*^G434V^, 5.41 ± 1.54 pA/pF; *Kcnc3*^(1-465)^, 7.62 ± 1.22 pA/pF; empty vector, 4.59 ± 0.46 pA/pF; untransfected CHO, 4.58 ± 0.55 pA/pF. Unpaired *t* test. ***P = 0.0001. (*J*) Side view of Kv1/2 hybrid tetramer cartoon model (four α subunits were shown in green, pink, blue, and yellow). (*Right*) Enlargement shows glycine is surrounded by hydrophobic amino acids from cis S6 and trans S5 domains. The X-ray crystallographic coordinates and structure factor data were obtained from the Protein Data Bank (accession no. 2R9R).

Quantitative real-time PCR analysis confirmed mRNA for *Kcnc3* in multiple brain regions of WT mice, including the hippocampus (Fig. 3*A*). Moreover, Western blots using an antibody against the C terminus of KCNC3 (KCNC3 691–708) found similar levels of KCNC3 protein in the hippocampus of both WT and *Clueless* mice but not in offspring of another independent, gene-edited founder mouse (*Kcnc3^1-465^*) that produced a C-terminal truncated protein (Fig. 3*B* and *SI Appendix*, Fig. S8). These findings were supported by a protein stability assay showing that both KCNC3 and KCNC3 (G434V) have similar half-lives (T_1/2_) in transfected Chinese hamster ovary (CHO) cells (*Kcnc3* T_1/2_ = 1.9 h; *Kcnc3^G434V^* T_1/2_ = 2.3 h) (*SI Appendix*, Fig. S8), suggesting that the *Clueless* mutation does not alter the expression or the stability of KCNC3 protein. Finally, immunohistochemical analysis revealed that KCNC3 and KCNC3 (G434V) are highly enriched in hippocampal mossy fibers (MFs) in both WT and *Clueless* mice (Fig. 3 *C* and *D*).

KCNC3 is an A-type, rapidly inactivating potassium channel that regulates action potential (AP) repolarization and is responsible for high-frequency firing in many neuron types (42–44). Each subunit of the KCNC3 tetramer consists of six transmembrane domains, of which S1 to S4 make up the voltage sensor domain and S5 and S6 form the pore. The G434 residue is located within a phylogenetically conserved region in S4 that is conserved across multiple species (Fig. 3 *E* and *F*). To investigate whether G434V alters membrane properties in vitro, we cotransfected CHO cells with full-length *Kcnc3^WT^*, mutant *Kcnc3^G434V^*, or truncated *Kcnc3^1-465^* constructs along with a green fluorescent protein (GFP) vector. Immunostaining showed that both KCNC3 and KCNC3 (G434V) were targeted to the cell surface of transfected cells (*SI Appendix*, Fig. S8).

Furthermore, voltage-clamp recordings of GFP-expressing cells showed an outwardly rectifying current in cells transfected with a *Kcnc3^WT^* construct (Fig. 3 *G* and *H*). Consistent with previous reports, recordings from 50% of cells transfected with the WT channel displayed inactivation currents (44). However, cells transfected with either *Kcnc3^G434V^* or *Kcnc3^1-465^* constructs manifested minimal outward currents (Fig. 3 *G* and *H*) with a significant reduction in current density (Fig. 3*I*). These findings are further supported by a structural model for voltage-gated potassium channels (45, 46). Based on the model, the corresponding glycine (G434) is surrounded by hydrophobic amino acids from both cis S6 and trans S5 domains. Replacing the glycine with a more hydrophobic valine could impair channel opening by increasing subunit interactions and the rigidity of this interface. This, in turn, could impair either channel gating or channel opening (Fig. 3*J* and *SI Appendix*, Fig. S8). Taken together, these results suggest that the *Kcnc3^G434V^* mutation impairs potassium conductance without altering KCNC3 protein expression.

### The *Clueless* Mutation Alters Firing Dynamics of Hippocampal Granule Cells.

**Fig. 4.**
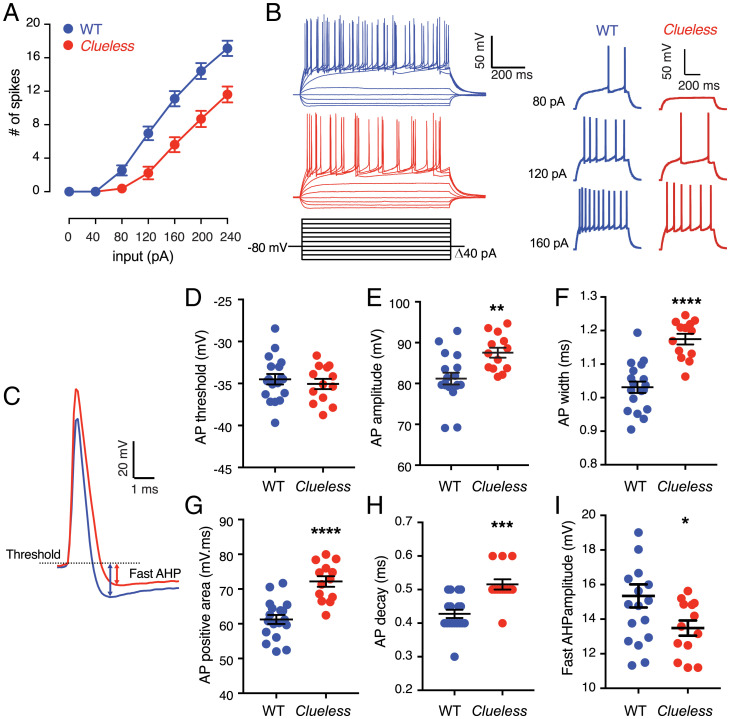
Characterization of intrinsic excitability of *Clueless* mutants in ex vivo adult hippocampal slices. (*A*) Granule cells from *Clueless* exhibit reduced AP firing. Two-way repeated measures ANOVA results for the following: genotype × input interaction, *F*_6,174_ =13.61, ****P < 0.0001; genotype effect, *F*_1,29_ =17.82, ***P = 0.0002. (*B*, *Left*) Superimposed sample traces from WT and *Clueless* dentate granule cells. (*B*, *Right*) Enlargements of sample traces at 80, 120, and 160 pA from WT and *Clueless* mice. (*C*) Superimposed sample traces of AP in granule cells in the hippocampal slices of 5- to 6-wk-old WT and *Clueless* mice. Granule cells from *Clueless* showed no change in (*D*) AP threshold but had (*E*) larger AP amplitude, (*F*) increased width, (*G*) larger AP-positive area, (*H*) longer decay time, and (*I*) smaller *f*AHP amplitude. WT (*n* = 18), *Clueless* (*n* = 13) from three mice per genotype. Unpaired Student’s *t* test. ****P < 0.0001; ***P < 0.001, **P < 0.01). Data are reported as mean ± SEM.

Given the high expression of KCNC3 protein in MFs and *Kcnc3* mRNA expression in the DG (Fig. 3 *C* and *D* and *SI Appendix*, Fig. S7) and the role for DG granule cells in spatial learning and memory (12, 39), we conducted whole-cell recording of DG granule cells in mouse brain slices. We found that DG granule cells from *Clueless* mice exhibited decreased neuronal firing compared with from WT mice (Fig. 4 *A* and *B*), in agreement with the reported role of KCNC3 in AP firing frequency (47, 48). Analysis of the AP waveform (Fig. 4*C*) revealed no significant differences in AP threshold (Fig. 4*D*) or rise time (WT, 0.26 ± 0.01 ms; *Clueless*, 0.26 ± 0.01 ms); however, DG granule cells from *Clueless* mice had a larger AP amplitude and width (Fig. 4 *E* and *F*), a larger AP-positive area (Fig. 4*G*) as a result of a longer AP decay (Fig. 4*H*), and a smaller fast after-hyperpolarization (*f*AHP) (Fig. 4*I*). The measured *f*AHP is consistent with the function of fast-inactivating potassium channels in hippocampal MFs (49). Furthermore, we observed an increased paired-pulse facilitation in the *Clueless* MF–CA3 synapses (*SI Appendix*, Fig. S9), suggesting a lower probability of neurotransmitter release from presynaptic granule cells (50). This may be due, at least in part, to the regulatory role that KCNC3 plays in mediating vesicle endocytosis in hippocampal neurons (51). Together with our in vitro results, these data show that *Clueless* reduces the KCNC3 voltage-dependent potassium current leading to prolonged spike repolarization and reduced AP firing in hippocampal granule cells, likely reducing the probability of neurotransmitter release at the MF synapses of *Clueless* mice and leading to their spatial-learning deficits (48, 49).

## Discussion

Our studies have uncovered a critical role for *Kcnc3* in spatial learning, as a missense mutation in this gene alters potassium currents through KCNC3 (K_v_3.3) channels. This leads to impairments in action-potential firing and the inhibition of *Npas4* target gene expression, ultimately reducing hippocampal-dependent learning and memory. Although the mutation (G434V) was initially discovered in an unbiased screen, the associated phenotypes were recapitulated in a genetically engineered *Kcnc3^G434V^* mutant, strongly suggesting that this specific mutation causes the learning and memory deficits.

 Previous studies on *Kcnc3* null mice noted only very mild gait phenotypes (52, 53). KCNC3 forms heterotetrametric complexes with other KCNC subunits to assemble into channels, and all four mammalian KCNC subunits are expressed in hippocampus (42, 54, 55). Therefore, knockout models may have been uninformative about the role of the KCNC3 channel in learning and memory, due to developmental compensation or functional redundancy. To address issues with redundancy, *Kcnc1*/*Kcnc3* double-null mice have been generated (52). While these mice have severe cerebellar motor defects and sleep abnormalities, they have no learning defects in an active avoidance task (52, 53, 56–58). However, it is possible that the type of learning phenotype that we describe could have been missed with the focus on cerebellar defects in the double-knockout mice. Taken together, it is clear that disruption of potassium channels can have pleiotropic effects, making full phenotypic characterization of null mutants a complex undertaking (59).

Similarly, transcriptomics approaches to find molecular markers for learning and memory have not indicated a specific role for *Kcnc3* (17, 19), even though *Kcnc3* gene expression increases in DG granule neurons after pentylenetetrazol activation (18). *Kcnc3* gene expression was also found to be altered in mice lacking another gene, *Adar3*, which is involved in RNA editing (60). Interestingly, although they also have other anxiety phenotypes, mice lacking functional *Adar3* have impaired responses in a fear-conditioning task (60), similar to our behavioral findings.

Our in vitro and electrophysiological data indicate that the KCNC3 (G434V) mutation reduces potassium currents generated by KCNC channels and influences neuronal excitability, thereby providing a cellular basis for the spatial-learning defects. Voltage-gated potassium channels play key roles in the electrical properties of neurons, and many of them are expressed in the hippocampus. However, only Kv4.2 (KCND2) and Kv12.2 (KCNH3) have been previously implicated in learning and memory (61–68).

In humans, mutations in *KCNC3* have been associated with spinocerebellar ataxia as well as hearing loss (69–71), including an early-onset form of spinocerebellar ataxia with severe intellectual disability (72). While our mouse mutants displayed a mild tremor, they had no gross motor or hearing deficits, suggesting that the G434V mutation did not alter these functions of the Kv3.3 channel. Nonetheless, it is unclear whether the learning phenotype in our mouse model is due to altered brain development or whether the mice would develop additional cerebellar phenotypes with age. It is also important to note that many of the human mutations tend to alter trafficking of the protein or increase potassium conductance (57), whereas the G434V mutation had no effect on protein expression.

In summary, our studies support the idea that forward genetics can serve as a powerful means to study complex behaviors such as learning and memory in mice. Mutations arising from forward genetic methods provide a complimentary strategy to reverse genetics, as they allow for the discovery of silencing mutations and hypomorphic alleles (73). Moreover, studies of *Clueless* and other ENU mutants have the potential to reveal novel regulators of learning and memory and new therapeutic targets for impairments of learning and memory.

## Materials and Methods

### Mutagenesis and Breeding Scheme to Produce Mutants.

WT C57BL/6J mice (stock no. 000664) were purchased from the Jackson Laboratory (Bar Harbor, ME). To induce mutations, 8- to 10-wk-old male C57BL/6J mice were intraperitoneally injected with 100 mg/kg ENU (Sigma N3385) once per week for three consecutive weeks (Fig. 1*A*) (34). Six weeks after injection, ENU-treated mice were mated with WT C57BL/6J females to produce G1 offspring. G1 males were bred with WT C57BL/6J females to produce G2 offspring. G2 females were backcrossed with their G1 sire to produce G3 progeny for both dominant and recessive phenotypic screening (34). Pedigrees that showed abnormal amount of freezing time in the contextual fear-conditioning test were maintained by intercrossing of offspring with extreme phenotypes. All ENU-mutagenized mice were generated at Northwestern University, following guidelines for animal care and use, and later were maintained at University of Texas (UT) Southwestern (UTSW) Medical Center in accordance with Institutional Animal Care and Use Committee (IACUC) guidelines. Mice were supplied with food and water ad libitum under a 12-h light:12-h dark cycle with controlled temperature and humidity. All experimental procedures were approved by the UTSW Medical Center IACUC.

### Genetic Mapping.

To control for the genetic background in mapping crosses, both WT and *Clueless* mutant F2 crosses were performed. For the WT control cross, WT C57BL/6J males were mated to C57BL/10J females (Jackson Laboratory, stock no.000665) to produce F1 (WTB10B6F1) mice, and F1 mice were further intercrossed to produce control WT F2 mapping mice (WTB10B6F2). A total of 264 WTB10B6F2 mice were involved in QTL mapping. To map mutants, *Clueless* males were mated to C57BL/10J females to produce F1 mice. F1 mice with low freezing scores were intercrossed to create F2 mice. Six F2 families (259 mice in total) with significant low freezing scores were used in genetic mapping. Genomic DNA from tails was prepared by phenol/chloroform/isoamyl alcohol extraction. Taqman probes for SNPs were designed by ABI (ThermoFisher, Foster City, CA) and tested on the BioMark HD Real-Time PCR System (Fluidigm, South Francisco, CA). All genotypes were performed on 96 × 96 genotyping plates, and SNP results were analyzed with Fluidigm SNP genotyping analysis software. The QTL analysis was performed with the R/QTL package (74). Interval mapping was performed using the scanone function with multiple imputation method (*n* = 1,000 imputations). The thresholds for LOD scores were determined with an empirical *P* value from 10,000 permutations.

### Exome Sequencing.

Sequencing was performed by the UTSW McDermott Center Next-Generation Sequencing Core, and data were analyzed by staff of the UTSW Bioinformatics Core Facility. In brief, exomes were captured with SureSelect Mouse All Exon Kit (Agilent, Santa Clara, CA), sequenced with ∼100× coverage, and variants were called with the Genome Analysis Toolkit (GATK) (75).

### Behavior Testing.

All behavioral experiments were completed during the light phase and mice were acclimated for 1 h before tests. Except for the G3 screening, at least two different cohorts of animals were tested for each behavior experiment. Behavioral scoring was performed without knowledge of genotypes. Method details for specific behavioral tests can be found in *SI Appendix*.

### *Production of Kcnc3^G434V^ mice by CRISPR/Cas9*.

Guide RNA was designed and synthesized by Sigma (5′-ACGCAGCCCCACGAAGTGACGG-3′). Donor oligonucleotide (200-nucleotide single-strand DNA) was designed by Sigma and synthesized by Integrated DNA Technologies. The donor DNA contains the G > T transition to create the *Clueless* mutation, C > T transition at 14 nt upstream of the *Clueless* mutation to block the PAM site and to create a Hinfl restriction site. The donor DNA contains a 92-nt homology arm at the 5′ end and a 93-nt homology arm at the 3′ end. C57BL/6J embryos were injected by staff at the UTSW Transgenic Core Facility, and founder mice were screened with Hinf1 (New England Biolabs, Ipswich, MA) digestion and Sanger sequencing with the primers described in the *Kcnc3^G434V^* mutation genotyping information. Positive founders were crossed with C57BL/6J mice to produce F1 offspring. Heterozygous F1 identified by Sanger sequencing were intercrossed to produce males for behavior tests. Two independent positive lines were kept (*Kcnc3_L50* and *Kcnc3_L53*). Another founder line that is missing one nucleotide around the *Clueless* mutation and leads to a stop codon nearby was also identified. This line produces a truncated KCNC3 protein (KCNC 1–465) which does not contain the intracellular C-terminal sequence.

### Stranded mRNA-Seq Library Preparation and Sequencing.

One hour after standard contextual fear-conditioning training, mice were euthanized by cervical dislocation and hippocampi were dissected on ice. Under the microscope, the dorsal and ventral parts of DG and CA were dissected in ice-cold RNAlater and then snap frozen in liquid nitrogen. The samples were stored at −80 °C. For each condition (i.e., WT with and without FCT; Clueless with and without FCT) there were four replicates. mRNAs were isolated using a Dynabead mRNA DIRECT Micro kit (Ambion) according to the manufacturer’s instructions. All mRNA samples were examined for quantity and quality by NanoDrop and Bioanalyzer 2100 (Agilent). Libraries were constructed following TruSeq Stranded mRNA Sample preparation guide (Illumina), and single-end 76mer sequencing was performed on a HiSEq. 2500 platform (Illumina) at the McDermott Sequencing Core at UTSW.

### Gene Set Enrichment.

Gene set enrichment analysis was performed using logistic regression accounting for gene length as a covariate (76). Heat maps were prepared showing odds ratio and false-discovery rate corrected *P* values for enrichment, if significant. For the gene set enrichment study, we curated a list of immediate early genes from the literature, as well as a list of putative NPAS4 target genes (40). As the background gene list of the gene set enrichment study, we used the 16,009 expressed genes in the hippocampus. Ensembl gene identifiers were converted to mouse genome informatics symbols using the *biomaRt* R package (77). The complete gene list used for the gene set enrichment is in Dataset S6. Additional details on data processing and differential gene expression analysis are provided in *SI Appendix*.

### Immunohistochemistry and Immunocytochemistry.

Eight-week-old male mice were anesthetized with 10 µL of Euthasol. When no reflex was observed, the mice were transcardially perfused with 0.1 M phosphate saline buffer (PBS; pH 7.4) and then 4% paraformaldehyde (PFA) in 0.1 M PBS (pH 7.4). The brains were postfixed in 4% PFA at 4 °C overnight and then cryoprotected in 30% sucrose in PBS. The brains were sectioned at 40 µm on a cryostat and processed with routine immunohistochemistry. In brief, the free-floating slices were rinsed with PBS twice and incubated with blocking buffer (10% donkey serum [Jackson ImmuneResearch], 0.3% Triton X-100 in PBS) for 1 h at room temperature to reduce nonspecific antibody binding and increase antibody penetration. The sections were then incubated with primary antibody (rabbit anti-KCNC3, 1:200; APC-102, Alomone Laboratories) in blocking buffer at 4 °C for overnight. The next day, the brain sections were washed with PBS three times and incubated with secondary antibody (donkey anti-rabbit Alexa 488, 1:1,000; ThermoFisher) in 10% donkey serum and PBS for 1 h. After three PBS washes, the sections were incubated with TO-PRO-3 Iodide (642/661) (1:100; ThermoFisher) in PBS for 1 h at room temperature. The sections were then rinsed with PBS and mounted with Prolong Gold Anti-Fade Reagent with DAPI on fluorescence-free glass slides (ThermoFisher). For immunocytochemistry, cells were fixed with 4% PFA in PBS for 10 min at room temperature and permeablized with 0.1% Triton X-100 in PBS for 10 min. Cells were then blocked with blocking buffer for 1 h and followed with the same procedure as immunohistochemistry.

### Western Blot.

Adult mouse brain tissues were homogenized using the FastPrep-24 beads system (MP Biomedicals) in 4 °C sucrose buffer (320 mM sucrose, 50 mM Tris⋅HCl, pH 7.4) with protease inhibitors (Sigma). After homogenization, 10× radioimmunoprecipitation assay buffer (RIPA) buffer (final concentration: 50 mM Tris⋅HCl, 150 mM NaCl, 5 mM ethylenediaminetetraacetic acid, 1% Triton, 1% sodium deoxycholate, 0.2% sodium dodecyl sulfate, pH 7.4) was added and the solution was rocked at 4 °C for 1 h. Then the samples were centrifuged at 12,000 × *g* for 10 min at 4 °C, and the supernatant was mixed with 6× sample buffer and incubated at 37 °C for 30 min. The proteins were separated on 7.5% sodium dodecyl sulfate–polyacrylamide gel electrophoresis gel and transferred to polyvinylidene difluoride (PVDF) membrane. Western blotting was performed according to standard protocols. In brief, the PVDF membrane was blocked with 5% nonfat dry milk in 0.1% triton in 0.1% triton in Tris-buffered saline (TBST) and then incubated with primary antibodies at 4 °C overnight (rabbit anti-KCNC3, 1:1,000, APC-102, Alomone Laboratories; β-actin mouse monoclonal antibody, 1:10,000, LI-COR, 926–42212). After wash with TBST, the PVDF membrane was incubated with secondary antibodies (goat anti-rabbit IRDye 680LT and goat anti-mouse IRDye 800CW, 1:20,000; LI-COR, 926–68021, 926–32210). Following washes, the membrane was scanned with the Odyssey imaging system (LI-COR). Rabbit anti-KCNC3 (APC-102) from Alomone Laboratories recognizes the intracellular C terminus of KCNC3, which is lost in KCNC3 (1–465). Tissues from *Kcnc3^(1-465)^* mice were used as negative controls for APC-102.

### Cell Culture, Transfection, and Protein Stability Assay.

CHO-K1 cells (Sigma, 85051005) were grown in Ham’s F-12 medium (ThermoFisher, 31765–092) supplemented with 10% fetal bovine serum (FBS) and penicillin-streptomycin (100 U/mL; ThermoFisher). At 80 to 90% confluency, the cells were cotransfected with 6 µg of pcDNA3 plasmids containing WT *Kcnc3*, *Kcnc3^G434V^*, and 1.2 µg of *eGFP* plasmids per 10-cm^2^ dish with Lipofectamine LTX (ThermoFisher, 15338030) following manufacturer’s instructions. Details on the vectors are provided in *SI Appendix*. The transfection was stopped 6 h later by medium change. At 24 h after transfection, cells were replated in 35-mm wells in triplicate. At 24 h after replating, cells were treated with 20 µg/mL cycloheximide (Sigma, C4859) for 0, 1, 2, 3, 4, and 5 h. At the indicated time, cells were lysed with RIPA buffer, and total protein was subjected to Western blot. The blots were scanned, and signals were measured with ImageStudioLite (LI-COR). The KCNC3 signal of each sample was adjusted with the actin signal, and the relative intensity compared with time 0 was calculated in all replicates to obtain the decay curve. The protein half-life was measured by the one phase exponential decay method in Prism 7 (GraphPad).

### Electrophysiology in CHO Cells.

CHO-K1 cells (Sigma, 85051005) were grown in Ham’s F-12 medium (ThermoFisher, 31765–092) supplemented with 10% FBS and penicillin-streptomycin (100 U/mL; ThermoFisher). At 80 to 90% confluency, the cells were cotransfected with 1 µg of pcDNA3 plasmids containing WT *Kcnc3*, *Kcnc3^G434V^*, truncated *Kcnc3^(1-465)^* and 0.25 µg of *eGFP* plasmid per 35 mm^2^ well with Lipofectamine LTX (ThermoFisher, 15338030) following manufacturer’s instructions. At 24 h after transfection, the cells were replated at low density in 24-well plates containing poly-d-lysine hydrobromide–coated glass coverslips (10 mg/mL; Sigma, P6407). Whole-cell currents in the transfected and untransfected CHO cells were recorded using a system including a Multiclamp 700B amplifier (Molecular Devices), Axon Digidata 1550 (Molecular Devices), and manipulator (MP-285, Sutter Instrument). The bath solution contained (in mM) 145 NaCl, 3 KCl, 10 HEPES, 3 CaCl_2_, 8 glucose, and 2 MgCl_2_ (pH was adjusted to 7.2 to 7.4 by NaOH, osmotic concentration = 290 to 300 Osmol/L). The internal solution contained (in mM): 125 potassium gluconate, 15 KCl, 10 HEPES, 4 MgATP, 0.3 Na-guanosine 5'-triphosphate (GTP), 10 Na-phosphocreatine, and 0.2 ethylene glycol tetraacetic acid (EGTA; pH 7.2 to 7.4; 290 to 300 mosM). The recording glass pipettes were made from borosilicate glass capillary (Sutter Instrument) by a micropipette puller P-97 (Sutter Instrument) and had resistances of 3 to 5 MΩ. All recordings were performed at room temperature. Cells were held at −70 mV and depolarized from −120 mV to 70 mV with 10-mV increments. Current density was calculated from peak potassium current (mean value, 20 ms window from the peak) divided by the capacitance of the cells we recorded. The data acquisition and analysis were performed with pClamp 10.4 software (Molecular Devices).

### Electrophysiology in Acutely Isolated Brain Slices.

Transverse hippocampal slices (350 μm) from 5- to 6-wk-old WT mice and *Clueless* mutant mice were obtained by cutting tangentially to the longitudinal axis of the hippocampus. The brain was sliced at 0 to 2 °C in artificial cerebrospinal fluid (ACSF) saturated with 95% O_2_/5% CO_2_ and containing (in mM): 119 NaCl, 2.5 KCl, 1.0 NaH_2_PO_4_, 4 MgSO_4_, 4 CaCl_2_, 26.2 NaHCO_3_, and 11 glucose. Slices recovered in a holding chamber for at least 1 h before use. During recordings, picrotoxin (100 μM) was added to block GABA_A_ receptor–mediated inhibitory postsynaptic currents (IPSCs).

### *CA3 pyramidal neurons*.

Whole-cell voltage-clamp recordings were performed as previously described (78). During recordings, slices were superfused with ACSF saturated with 95% O_2_/5% CO_2_ (at 23 °C) and containing (in mM): 119 NaCl, 2.5 KCl, 1.0 NaH_2_PO_4_, 4 MgSO_4_, 4 CaCl_2_, 26.2 NaHCO_3_, and 11 glucose. High Ca^2+^ and Mg^2+^ concentrations (4 mM) for extracellular solutions were used to reduce cellular excitability and thus to inhibit the epileptiform activity to which the CA3 region is especially prone (as performed by authors of refs. 79–81). It also allows better isolation of the MF-CA3 excitatory postsynaptic currents (EPSCs) from recurrent associational/commissural (i.e., polysynaptic) EPSCs. Pyramidal cells in the CA3 field were visualized using infrared-differential interference contrast optics. Synaptically evoked EPSCs were measured using a Multiclamp 700B amplifier (Molecular Devices, Foster City, CA). Recording electrodes (3 to 5 MΩ) contained (in mM): 120 Cs-gluconate, 20 KCl, 10 HEPES, 0.2 EGTA, 2 MgCl_2_, 4 MgATP, and 0.3 NaGTP. Afferents were stimulated at 0.1 Hz by a glass monopolar microelectrode filled with ACSF that was always positioned in the granular cell layer of the DG or in the DG hilus. For most of the experiments, LY-354740 1 μM, a highly selective and potent agonist of group II mGlu (metabotropic glutamate) receptors, was also applied at the end of the experiments to verify that evoked EPSCs were mediated by glutamate release from MF. Data were filtered at 2 kHz, digitized at 10 kHz, and collected and analyzed using Clampex 10.3 software (Clampex 10.3.0.2, Molecular Devices). Membrane potentials of CA3 neurons ranged between −75 and −65 mV. Series resistances ranged from 10 to 20 MΩ, and input resistances were monitored online with a 40 pA/150 ms current injection given before every stimulus. Only cells with a stable resistance (Δ < 15%) for the duration of the recording were kept for analysis. Recordings and analyses were performed blind regarding the genotype. Sample sizes are similar to those in standard electrophysiological studies conducted with rigor and reproducibility (i.e., sizes were chosen so that differences between groups can be interpreted in an unequivocal manner).

### *DG granule cells*.

Whole-cell current-clamp recordings were performed as previously described (82–84). During recordings, slices were superfused with ACSF (31.5 to 32.5 °C) saturated with 95% O_2_/5% CO_2_ and containing (in mM): 119 NaCl, 2.5 KCl, 1.0 NaH_2_PO_4_, 1.3 MgSO_4_, 2.5 CaCl_2_, 26.2 NaHCO_3_, and 11 glucose. To measure neuronal firing, whole-cell current-clamp recordings were performed with electrodes (3 to 5 MΩ) containing (in mM): 120 K-gluconate, 20 KCl, 10 HEPES, 0.2 EGTA, 2 MgCl_2_, 4 Na_2_ATP, and 0.3 Tris–GTP at a pH of 7.20 to 7.25. Data were filtered at 5 KHz, digitized at 10 kHz, collected and analyzed using Clampex 10.5 software (Clampex 10.5.0.9, Molecular Devices, Inc.). Membrane potentials were maintained at –80 mV, series resistances (10 to 18 MΩ), and input resistances were monitored online with a 40-pA current injection (150 ms) given before each 700-ms current injection stimulus. Only cells with a stable input resistance (Δ < 10%) for the duration of the recording were kept for analysis. The firing rate represents the average value measured from two to three cycles (700 ms duration; –120 to +240 pA range with a 40-pA step increment every 15 s). The resting membrane potential and the spike threshold were not adjusted for the liquid junction potential (∼8 mV). Spike characteristics were obtained from the first spike evoked by the minimal depolarizing current pulse in every granule cell recorded; the spike threshold was obtained by using the maximum of the third differential during the rising phase. The other characteristics were measured as follows: spike amplitude, difference between spike threshold and peak; spike width, measured at 20 pA above threshold; *f*AHP amplitude, difference between spike threshold and maximum hyperpolarization after the spike; spike rise, time difference between 10 and 90% of the spike peak; decay, time from 90 to 37% of AP repolarization phase; AP-positive area, AP area above threshold; peak-to-AHP, time from AP peak to 90% of AHP.

### Quantification and Statistical Analysis.

Unless otherwise stated, all statistical analyses were performed with Prism 7 (GraphPad). Two-group comparisons were performed with Kolmogorov-Smirnov test or unpaired Student’s *t* test when appropriate. Multiple-group comparisons were performed with one-way or two-way ANOVA followed by post hoc test when appropriate. Data are presented as mean ± SD and mean ± SEM when appropriate.

## Supplementary Material

Supplementary File

Supplementary File

Supplementary File

Supplementary File

Supplementary File

## Data Availability

RNA-sequencing data have been deposited in the National Center for Biotechnology Information Gene Expression Omnibus repository (accession no. GSE202094)(85). All other data are included in the article and/or supporting information. The deposited data will be accessible upon publication in https://www.ncbi.nlm.nih.gov/geo/query/acc.cgi?acc=GSE202094.

## References

[1] C. M. Alberini, E. R. Kandel, The regulation of transcription in memory consolidation. Cold Spring Harb. Perspect. Biol. 7, a021741 (2014). 25475090 10.1101/cshperspect.a021741 PMC4292167

[2] E. R. Kandel, Y. Dudai, M. R. Mayford, The molecular and systems biology of memory. Cell 157, 163–186 (2014). 24679534 10.1016/j.cell.2014.03.001

[3] G. R. Owen, E. A. Brenner, Mapping molecular memory: Navigating the cellular pathways of learning. Cell. Mol. Neurobiol. 32, 919–941 (2012). 22488526 10.1007/s10571-012-9836-0 PMC11498452

[4] A. J. Silva, Y. Zhou, T. Rogerson, J. Shobe, J. Balaji, Molecular and cellular approaches to memory allocation in neural circuits. Science 326, 391–395 (2009). 19833959 10.1126/science.1174519 PMC2844777

[5] S. Tonegawa, X. Liu, S. Ramirez, R. Redondo, Memory engram cells have come of age. Neuron 87, 918–931 (2015). 26335640 10.1016/j.neuron.2015.08.002

[6] R. A. Nicoll, A brief history of long-term potentiation. Neuron 93, 281–290 (2017). 28103477 10.1016/j.neuron.2016.12.015

[7] T. Kitamura , Engrams and circuits crucial for systems consolidation of a memory. Science 356, 73–78 (2017). 28386011 10.1126/science.aam6808 PMC5493329

[8] J. C. Kaldun, S. G. Sprecher, Initiated by CREB: Resolving gene regulatory programs in learning and memory: Switch in cofactors and transcription regulators between memory consolidation and maintenance network. BioEssays 41, e1900045 (2019). 31237359 10.1002/bies.201900045

[9] S. Tonegawa, M. D. Morrissey, T. Kitamura, The role of engram cells in the systems consolidation of memory. Nat. Rev. Neurosci. 19, 485–498 (2018). 29970909 10.1038/s41583-018-0031-2

[10] D. O. Seo, L. E. Motard, M. R. Bruchas, Contemporary strategies for dissecting the neuronal basis of neurodevelopmental disorders. Neurobiol. Learn. Mem. 165, 106835 (2019). 29550367 10.1016/j.nlm.2018.03.015 PMC6138573

[11] A. R. Garner , Generation of a synthetic memory trace. Science 335, 1513–1516 (2012). 22442487 10.1126/science.1214985 PMC3956300

[12] X. Liu , Optogenetic stimulation of a hippocampal engram activates fear memory recall. Nature 484, 381–385 (2012). 22441246 10.1038/nature11028 PMC3331914

[13] S. Ramirez , Creating a false memory in the hippocampus. Science 341, 387–391 (2013). 23888038 10.1126/science.1239073

[14] D. S. Roy , Distinct neural circuits for the formation and retrieval of episodic memories. Cell 170, 1000–1012.e19 (2017). 28823555 10.1016/j.cell.2017.07.013 PMC5586038

[15] M. B. Chen, X. Jiang, S. R. Quake, T. C. Südhof, Persistent transcriptional programmes are associated with remote memory. Nature 587, 437–442 (2020). 33177708 10.1038/s41586-020-2905-5 PMC9097329

[16] A. Katzman , Distinct transcriptomic profiles in the dorsal hippocampus and prelimbic cortex are transiently regulated following episodic learning. J. Neurosci. 41, 2601–2614 (2021). 33536202 10.1523/JNEUROSCI.1557-20.2021 PMC8018728

[17] G. Hermey , Genome-wide profiling of the activity-dependent hippocampal transcriptome. PLoS One 8, e76903 (2013). 24146943 10.1371/journal.pone.0076903 PMC3798291

[18] B. Lacar , Nuclear RNA-seq of single neurons reveals molecular signatures of activation. Nat. Commun. 7, 11022 (2016). 27090946 10.1038/ncomms11022 PMC4838832

[19] P. Rao-Ruiz , Engram-specific transcriptome profiling of contextual memory consolidation. Nat. Commun. 10, 2232 (2019). 31110186 10.1038/s41467-019-09960-x PMC6527697

[20] S. G. Grant , Impaired long-term potentiation, spatial learning, and hippocampal development in fyn mutant mice. Science 258, 1903–1910 (1992). 1361685 10.1126/science.1361685

[21] A. J. Silva, R. Paylor, J. M. Wehner, S. Tonegawa, Impaired spatial learning in alpha-calcium-calmodulin kinase II mutant mice. Science 257, 206–211 (1992). 1321493 10.1126/science.1321493

[22] A. J. Silva , Alpha calcium/calmodulin kinase II mutant mice: Deficient long-term potentiation and impaired spatial learning. Cold Spring Harb. Symp. Quant. Biol. 57, 527–539 (1992). 1339689 10.1101/sqb.1992.057.01.058

[23] M. Mayford, E. R. Kandel, Genetic approaches to memory storage. Trends Genet. 15, 463–470 (1999). 10529810 10.1016/s0168-9525(99)01846-6

[24] A. Matynia , A high through-put reverse genetic screen identifies two genes involved in remote memory in mice. PLoS One 3, e2121 (2008). 18464936 10.1371/journal.pone.0002121 PMC2373872

[25] Y. S. Lee, Genes and signaling pathways involved in memory enhancement in mutant mice. Mol. Brain 7, 43 (2014). 24894914 10.1186/1756-6606-7-43 PMC4050447

[26] J. S. Takahashi, L. H. Pinto, M. H. Vitaterna, Forward and reverse genetic approaches to behavior in the mouse. Science 264, 1724–1733 (1994). 8209253 10.1126/science.8209253 PMC3830945

[27] M. Bućan, T. Abel, The mouse: Genetics meets behaviour. Nat. Rev. Genet. 3, 114–123 (2002). 11836505 10.1038/nrg728

[28] E. M. Moresco, X. Li, B. Beutler, Going forward with genetics: Recent technological advances and forward genetics in mice. Am. J. Pathol. 182, 1462–1473 (2013). 23608223 10.1016/j.ajpath.2013.02.002 PMC3644711

[29] Y. Wang , Large-scale forward genetics screening identifies Trpa1 as a chemosensor for predator odor-evoked innate fear behaviors. Nat. Commun. 9, 2041 (2018). 29795268 10.1038/s41467-018-04324-3 PMC5966455

[30] Y. Dudai, Y. N. Jan, D. Byers, W. G. Quinn, S. Benzer, dunce, a mutant of *Drosophila* deficient in learning. Proc. Natl. Acad. Sci. U.S.A. 73, 1684–1688 (1976). 818641 10.1073/pnas.73.5.1684 PMC430364

[31] W. G. Quinn, P. P. Sziber, R. Booker, The *Drosophila* memory mutant amnesiac. Nature 277, 212–214 (1979). 121760 10.1038/277212a0

[32] M. S. Livingstone, P. P. Sziber, W. G. Quinn, Loss of calcium/calmodulin responsiveness in adenylate cyclase of rutabaga, a *Drosophila* learning mutant. Cell 37, 205–215 (1984). 6327051 10.1016/0092-8674(84)90316-7

[33] T. Tully, Discovery of genes involved with learning and memory: An experimental synthesis of Hirschian and Benzerian perspectives. Proc. Natl. Acad. Sci. U.S.A. 93, 13460–13467 (1996). 8942957 10.1073/pnas.93.24.13460 PMC33631

[34] S. M. Siepka, J. S. Takahashi, Forward genetic screens to identify circadian rhythm mutants in mice. Methods Enzymol. 393, 219–229 (2005). 15817290 10.1016/S0076-6879(05)93007-3 PMC3757086

[35] L. G. Reijmers , A mutant mouse with a highly specific contextual fear-conditioning deficit found in an *N*-ethyl-*N*-nitrosourea (ENU) mutagenesis screen. Learn. Mem. 13, 143–149 (2006). 16585790 10.1101/lm.98606 PMC1409825

[36] V. Kumar , C57BL/6N mutation in cytoplasmic FMRP interacting protein 2 regulates cocaine response. Science 342, 1508–1512 (2013). 24357318 10.1126/science.1245503 PMC4500108

[37] P. Tovote, J. P. Fadok, A. Lüthi, Neuronal circuits for fear and anxiety. Nat. Rev. Neurosci. 16, 317–331 (2015). 25991441 10.1038/nrn3945

[38] T. Murai, S. Okuda, T. Tanaka, H. Ohta, Characteristics of object location memory in mice: Behavioral and pharmacological studies. Physiol. Behav. 90, 116–124 (2007). 17049363 10.1016/j.physbeh.2006.09.013

[39] C. A. Denny , Hippocampal memory traces are differentially modulated by experience, time, and adult neurogenesis. Neuron 83, 189–201 (2014). 24991962 10.1016/j.neuron.2014.05.018 PMC4169172

[40] B. L. Bloodgood, N. Sharma, H. A. Browne, A. Z. Trepman, M. E. Greenberg, The activity-dependent transcription factor NPAS4 regulates domain-specific inhibition. Nature 503, 121–125 (2013). 24201284 10.1038/nature12743 PMC4169177

[41] N. Hashikawa-Hobara, S. Mishima, C. Okujima, Y. Shitanishi, N. Hashikawa, Npas4 impairs fear memory via phosphorylated HDAC5 induced by CGRP administration in mice. Sci. Rep. 11, 7006 (2021). 33772088 10.1038/s41598-021-86556-w PMC7997869

[42] B. Rudy, C. J. McBain, Kv3 channels: Voltage-gated K+ channels designed for high-frequency repetitive firing. Trends Neurosci. 24, 517–526 (2001). 11506885 10.1016/s0166-2236(00)01892-0

[43] Y. Zhang , Kv3.3 channels bind Hax-1 and Arp2/3 to assemble a stable local actin network that regulates channel gating. Cell 165, 434–448 (2016). 26997484 10.1016/j.cell.2016.02.009 PMC4826296

[44] F. R. Fernandez, E. Morales, A. J. Rashid, R. J. Dunn, R. W. Turner, Inactivation of Kv3.3 potassium channels in heterologous expression systems. J. Biol. Chem. 278, 40890–40898 (2003). 12923191 10.1074/jbc.M304235200

[45] S. B. Long, X. Tao, E. B. Campbell, R. MacKinnon, Atomic structure of a voltage-dependent K+ channel in a lipid membrane-like environment. Nature 450, 376–382 (2007). 18004376 10.1038/nature06265

[46] L. Y. Jan, Y. N. Jan, Voltage-gated potassium channels and the diversity of electrical signalling. J. Physiol. 590, 2591–2599 (2012). 22431339 10.1113/jphysiol.2011.224212 PMC3424718

[47] P. D. Dodson, I. D. Forsythe, K. Presynaptic, Presynaptic K+ channels: Electrifying regulators of synaptic terminal excitability. Trends Neurosci. 27, 210–217 (2004). 15046880 10.1016/j.tins.2004.02.012

[48] L. K. Kaczmarek, Y. Zhang, Kv3 channels: Enablers of rapid firing, neurotransmitter release, and neuronal endurance. Physiol. Rev. 97, 1431–1468 (2017). 28904001 10.1152/physrev.00002.2017 PMC6151494

[49] J. R. Geiger, P. Jonas, Dynamic control of presynaptic Ca(2+) inflow by fast-inactivating K(+) channels in hippocampal mossy fiber boutons. Neuron 28, 927–939 (2000). 11163277 10.1016/s0896-6273(00)00164-1

[50] D. Fioravante, W. G. Regehr, Short-term forms of presynaptic plasticity. Curr. Opin. Neurobiol. 21, 269–274 (2011). 21353526 10.1016/j.conb.2011.02.003 PMC3599780

[51] X. S. Wu , Presynaptic Kv3 channels are required for fast and slow endocytosis of synaptic vesicles. Neuron 109, 938–946.e5 (2021). 33508244 10.1016/j.neuron.2021.01.006 PMC7979485

[52] F. Espinosa , Alcohol hypersensitivity, increased locomotion, and spontaneous myoclonus in mice lacking the potassium channels Kv3.1 and Kv3.3. J. Neurosci. 21, 6657–6665 (2001). 11517255 10.1523/JNEUROSCI.21-17-06657.2001 PMC6763102

[53] R. H. Joho, C. Street, S. Matsushita, T. Knöpfel, Behavioral motor dysfunction in Kv3-type potassium channel-deficient mice. Genes Brain Behav. 5, 472–482 (2006). 16923152 10.1111/j.1601-183X.2005.00184.x

[54] M. Weiser , Differential expression of Shaw-related K+ channels in the rat central nervous system. J. Neurosci. 14, 949–972 (1994). 8120636 10.1523/JNEUROSCI.14-03-00949.1994 PMC6577540

[55] S. Y. Chang , Distribution of Kv3.3 potassium channel subunits in distinct neuronal populations of mouse brain. J. Comp. Neurol. 502, 953–972 (2007). 17444489 10.1002/cne.21353

[56] F. Espinosa, G. Marks, N. Heintz, R. H. Joho, Increased motor drive and sleep loss in mice lacking Kv3-type potassium channels. Genes Brain Behav. 3, 90–100 (2004). 15005717 10.1046/j.1601-183x.2003.00054.x

[57] Y. Zhang, L. K. Kaczmarek, Kv3.3 potassium channels and spinocerebellar ataxia. J. Physiol. 594, 4677–4684 (2016). 26442672 10.1113/JP271343 PMC4983625

[58] F. Espinosa, M. A. Torres-Vega, G. A. Marks, R. H. Joho, Ablation of Kv3.1 and Kv3.3 potassium channels disrupts thalamocortical oscillations in vitro and in vivo. J. Neurosci. 28, 5570–5581 (2008). 18495891 10.1523/JNEUROSCI.0747-08.2008 PMC3844809

[59] C. S. Ho, R. W. Grange, R. H. Joho, Pleiotropic effects of a disrupted K+ channel gene: Reduced body weight, impaired motor skill and muscle contraction, but no seizures. Proc. Natl. Acad. Sci. U.S.A. 94, 1533–1538 (1997). 9037088 10.1073/pnas.94.4.1533 PMC19826

[60] D. Mladenova , Adar3 is involved in learning and memory in mice. Front. Neurosci. 12, 243 (2018). 29719497 10.3389/fnins.2018.00243 PMC5914295

[61] A. Miyake , Disruption of the ether-a-go-go K+ channel gene BEC1/KCNH3 enhances cognitive function. J. Neurosci. 29, 14637–14645 (2009). 19923296 10.1523/JNEUROSCI.0901-09.2009 PMC6665833

[62] X. Chen , Deletion of Kv4.2 gene eliminates dendritic A-type K+ current and enhances induction of long-term potentiation in hippocampal CA1 pyramidal neurons. J. Neurosci. 26, 12143–12151 (2006). 17122039 10.1523/JNEUROSCI.2667-06.2006 PMC6675426

[63] J. N. Lugo, A. L. Brewster, C. M. Spencer, A. E. Anderson, Kv4.2 knockout mice have hippocampal-dependent learning and memory deficits. Learn. Mem. 19, 182–189 (2012). 22505720 10.1101/lm.023614.111 PMC3348517

[64] J. Vernon, E. E. Irvine, M. Peters, J. Jeyabalan, K. P. Giese, Phosphorylation of K+ channels at single residues regulates memory formation. Learn. Mem. 23, 174–181 (2016). 26980786 10.1101/lm.040816.115 PMC4793203

[65] A. W. Varga, A. E. Anderson, J. P. Adams, H. Vogel, J. D. Sweatt, Input-specific immunolocalization of differentially phosphorylated Kv4.2 in the mouse brain. Learn. Mem. 7, 321–332 (2000). 11040264 10.1101/lm.35300 PMC311342

[66] K. P. Giese , Reduced K+ channel inactivation, spike broadening, and after-hyperpolarization in Kvbeta1.1-deficient mice with impaired learning. Learn. Mem. 5, 257–273 (1998). 10454353 PMC311244

[67] A. C. Need, E. E. Irvine, K. P. Giese, Learning and memory impairments in Kv beta 1.1-null mutants are rescued by environmental enrichment or ageing. Eur. J. Neurosci. 18, 1640–1644 (2003). 14511342 10.1046/j.1460-9568.2003.02889.x

[68] B. Rudy , “Voltage gated potassium channels: Structure and function of Kv1 to Kv9 subfamilies” in Encyclopedia of Neuroscience, L. R. Squire, Ed. (Academic Press, 2009), pp. 397–425.

[69] C. Gallego-Iradi , KCNC3(R420H), a K(+) channel mutation causative in spinocerebellar ataxia 13 displays aberrant intracellular trafficking. Neurobiol. Dis. 71, 270–279 (2014). 25152487 10.1016/j.nbd.2014.08.020 PMC4181561

[70] J. C. Middlebrooks , Mutation in the kv3.3 voltage-gated potassium channel causing spinocerebellar ataxia 13 disrupts sound-localization mechanisms. PLoS One 8, e76749 (2013). 24116147 10.1371/journal.pone.0076749 PMC3792041

[71] S. Khare , A KCNC3 mutation causes a neurodevelopmental, non-progressive SCA13 subtype associated with dominant negative effects and aberrant EGFR trafficking. PLoS One 12, e0173565 (2017). 28467418 10.1371/journal.pone.0173565 PMC5414954

[72] A. Duarri , Functional analysis helps to define KCNC3 mutational spectrum in Dutch ataxia cases. PLoS One 10, e0116599 (2015). 25756792 10.1371/journal.pone.0116599 PMC4355074

[73] J. S. Takahashi, K. Shimomura, V. Kumar, Searching for genes underlying behavior: Lessons from circadian rhythms. Science 322, 909–912 (2008). 18988844 10.1126/science.1158822 PMC3744585

[74] K. W. Broman, H. Wu, S. Sen, G. A. Churchill, R/qtl: QTL mapping in experimental crosses. Bioinformatics 19, 889–890 (2003). 12724300 10.1093/bioinformatics/btg112

[75] M. A. DePristo , A framework for variation discovery and genotyping using next-generation DNA sequencing data. Nat. Genet. 43, 491–498 (2011). 21478889 10.1038/ng.806 PMC3083463

[76] C. Lee, E. Y. Kang, M. J. Gandal, E. Eskin, D. H. Geschwind, Profiling allele-specific gene expression in brains from individuals with autism spectrum disorder reveals preferential minor allele usage. Nat. Neurosci. 22, 1521–1532 (2019). 31455884 10.1038/s41593-019-0461-9 PMC6750256

[77] S. Durinck, P. T. Spellman, E. Birney, W. Huber, Mapping identifiers for the integration of genomic datasets with the R/Bioconductor package biomaRt. Nat. Protoc. 4, 1184–1191 (2009). 19617889 10.1038/nprot.2009.97 PMC3159387

[78] A. Segev , Reduced GluN1 in mouse dentate gyrus is associated with CA3 hyperactivity and psychosis-like behaviors. Mol. Psychiatry 25, 2832–2843 (2020). 30038231 10.1038/s41380-018-0124-3 PMC6344327

[79] H. B. Kwon, P. E. Castillo, Role of glutamate autoreceptors at hippocampal mossy fiber synapses. Neuron 60, 1082–1094 (2008). 19109913 10.1016/j.neuron.2008.10.045 PMC4454280

[80] H. B. Kwon, P. E. Castillo, Long-term potentiation selectively expressed by NMDA receptors at hippocampal mossy fiber synapses. Neuron 57, 108–120 (2008). 18184568 10.1016/j.neuron.2007.11.024 PMC2390917

[81] D. L. Hunt, N. Puente, P. Grandes, P. E. Castillo, Bidirectional NMDA receptor plasticity controls CA3 output and heterosynaptic metaplasticity. Nat. Neurosci. 16, 1049–1059 (2013). 23852115 10.1038/nn.3461 PMC3740388

[82] S. Kourrich, J. R. Klug, M. Mayford, M. J. Thomas, AMPAR-independent effect of striatal αCaMKII promotes the sensitization of cocaine reward. J. Neurosci. 32, 6578–6586 (2012). 22573680 10.1523/JNEUROSCI.6391-11.2012 PMC3448780

[83] S. Kourrich, M. J. Thomas, Similar neurons, opposite adaptations: Psychostimulant experience differentially alters firing properties in accumbens core versus shell. J. Neurosci. 29, 12275–12283 (2009). 19793986 10.1523/JNEUROSCI.3028-09.2009 PMC3307102

[84] S. Kourrich , Dynamic interaction between sigma-1 receptor and Kv1.2 shapes neuronal and behavioral responses to cocaine. Cell 152, 236–247 (2013). 23332758 10.1016/j.cell.2012.12.004 PMC4159768

[85] Xu ., data available in NCBI Gene Expression Omnibus, https://www.ncbi.nlm.nih.gov/geo/query/acc.cgi?acc=GSE202094, deposited May 2022.

