# Supplementary material for: A missense mutation in Kcnc3 causes hippocampal learning deficits in mice
Source: Proc Natl Acad Sci U S A. 2022 Jul 26;119(31):e2204901119. doi: 10.1073/pnas.2204901119 (PMC9351536; doi:10.1073/pnas.2204901119)
Supplement: Supplementary File [file pnas.2204901119.sapp.pdf]

PNAS

## Supplementary Information for

### A Missense Mutation in *Kcnc3* Causes Hippocampal Learning Deficits in Mice

Pin Xu[1], Kazuhiro Shimomura[2], Changhoon Lee[1], Xiaofei Gao[3], Eleanor H. Simpson[4,5], Guocun Huang[1], Chryshanthi M. Joseph[1], Vivek Kumar[1,13], Woo-Ping Ge[1,3,6], Karen S. Pawlowski[6], Mitchell D. Frye[7], Saïd Kourrich[1,7, 14], Eric R. Kandel[4,8,9,10,11], Joseph S. Takahashi[1,12*]

[1]Department of Neuroscience, University of Texas Southwestern Medical Center, Dallas, TX 75390, USA.

[2]Department of Neurobiology, Northwestern University, Evanston, IL 60208, USA.

[3]Children's Medical Center Research Institute, University of Texas Southwestern Medical Center, TX 75390, USA.

[4]Department of Psychiatry, Columbia University, New York, NY 10032, USA.

[5]New York State Psychiatric Institute, New York, NY 10032, USA.

[6]Department of Pediatrics, University of Texas Southwestern Medical Center, Dallas, TX 75390-9152, USA.

[7]Department of Psychiatry, University of Texas Southwestern Medical Center, Dallas, TX 75390, USA.

[8]Department of Neuroscience, Columbia University, New York, NY 10032, USA.

[9]Kavli Institute of Brain Science, Columbia University, New York, NY 10032, USA.

[10] Mortimer B. Zuckerman Mind Brain Behavior Institute, Columbia University, New York, NY 10032, USA

[11]Howard Hughes Medical Institute, New York, NY 10032, USA.

[12]Howard Hughes Medical Institute, University of Texas Southwestern Medical Center, Dallas, TX 75390, USA.

[13]Present address: The Jackson Laboratory, Bar Harbor, ME 04609, USA.

[14]Present address: Centre d'Excellence en Recherche sur les Maladies Orphelines—Fondation Courtois (CERMO-FC) and Département des Sciences

Biologiques, Université du Québec à Montréal, Montréal, QC, Canada; and Center for Studies in Behavioral Neurobiology, Concordia University, Montreal, QC, Canada

*Corresponding author: Joseph S. Takahashi

**E-mail:** joseph.takahashi@utsouthwestern.edu

**This PDF file includes:**

Supplementary Text
Figures S1 to S10
Legends for Datasets S1 to S6
SI References

**Other supplementary materials for this manuscript include the following:**

Datasets S1 to S6

## Supplementary Information Text

### Behavior Testing

***Fear conditioning tests.*** For phenotypic screening, 7-8-week-old mice were trained on two consecutive days in the fear-conditioning chambers (Med Associate, Fairfax, VT). Mice were allowed to freely explore the chamber for 3 mins and then a tone (2.8 KHz, 85 dB) was applied for 30 secs which overlapped with a 2 second 0.85 mA foot shock at the end of the tone. Paired stimuli were applied 4 times each day with a 1-min intertrial interval. Twenty-four hrs after the second training day, mice were re-exposed to the context (5 min) followed by the sound cue (30 s). A stereotypical fear behavior, freezing, was scored. We defined freezing as the absence of movement except required for respiration (1, 2). We used the average % freezing time of day 2 (between the 2$^{nd}$ and the 3$^{rd}$ min) and day 3 (from the 2$^{nd}$ to 5$^{th}$ mins) to represent the contextual fear memory. For standard contextual and cued tests, mice were trained with the same protocol for one day, and both contextual (in the same chamber as trained) and cued (in a different chamber with different context and odor cues in another room) memory were tested 5 min (memory acquisition), 1 hr, or 24 hrs later (3). Separate cohorts of WT and mutant littermates were tested at each timepoint to avoid extinction effects. The shock intensity from each pair of neighboring wire bars on the grids was measured by an amp meter (Med Associates) and sound was calibrated with a sound meter. The metal grid floors (for training) and plastic floors (novel context) were washed with water, sprayed with 70% ethanol and dried before each trial to avoid odor cues. All sessions were videotaped and scored by FreezeFrame 3 (Actimetrics, Wilmette, IL).

***Foot shock intensity test.*** Naïve male mice (7-8 weeks old) were given different intensities of foot shocks in the fear conditioning chambers (from 0.05 mA to 0.40 mA with 0.05 mA increment steps). Shock intensity levels that evoked flinch, vocalization, and jumps were measured.

***Hearing sensitivity test.*** Auditory brainstem response (ABR) was performed on male mice (10-12 weeks old). The stimulus presentation, ABR acquisition, equipment control, and data management were coordinated using the computerized Intelligent Hearing Systems (IHS, Miami, FL, USA) SmartEP software (version 5.36). IHS6699 high frequency transducers coupled with the IHS system generated specific acoustic stimuli (tone bursts of different frequencies). Output was channeled into the ear canal through 10 cm long, 3 mm diameter plastic tubes. Calibrations [using a ½ inch microphone (Brüel & Kjær instruments, 4134) and a sound level meter with a fast Fourier transform (FFT) analyzer (Larson Davis 831)] were made with reference to the programmed output for 70 dB sound pressure levels (SPL re: 20 $\mu$Pa).

Briefly, the animals were anesthetized with tribromoethanol (i.p.), and placed on a T/Pump isothermal pad (Stryker Medical, model TP700, Portage, MI,

USA) in a sound-attenuating chamber (ETS-Lindgren Acoustic Systems, Cedar Park, TX, USA), maintaining body temperature at 37.5°C throughout testing. Subdermal neurology needle electrodes (Neuroline Subdermal, Ambu Inc., Columbia, MD, USA) were inserted at the vertex (+), ventrolateral to the left ear (-), and the left thigh (ground) to record responses. ABRs were elicited with tone bursts at 8, 16, and 32 kHz (0.5 ms rise/fall Blackman ramp, 1 ms duration, alternating phase) presented to the left ear of the animal at the rate of 21/s. ABRs were band-pass filtered below 100 Hz and above 3000 Hz, amplified, then computer averaged and displayed. The stimulus intensity was reduced in 10-20 dB steps and finally 5 dB steps to identify the lowest intensity at which a repeatable ABR waveform was detectable for each animal. ABR waveforms were generated from averages of 1052 stimuli for each stimulus condition, and data were stored digitally for later offline measurements and analyses.

***Olfactory discrimination test***. Mice were presented 5 different odors (water, lemon oil, vanilla oil, male odor and female odor in sequence) with 3 trials for a length of 2 mins per odor and the amount of sniffing time was recorded. The inter-trial interval was 1 min (4).

***Open field test.*** The open field used was a 55.8 cm x 55.8 cm x 35.6 cm (length x width x height) square arena with a white Plexiglas floor and white laminate walls (Phenome Technologies, Lincolnshire, IL). The behavior of 10 to 20-week-old males was observed and position in the arena over time was recorded using a video camera-based computer tracking system (Limelight 4, Actimetrics). Mice were then removed and returned to their home cages. The arena was cleaned with diluted *Quatricide* Detergent/Disinfectant (Pharmacal Research Labs, Waterbury, CT) between trials to avoid olfactory cues. The arena image was divided equally into 25 virtual zones (5 x 5). The 15 zones along the edge were defined as the peripheral region and the 10 zones in the middle were defined as the center region (5). The mice were allowed to explore the arena for 1 hr which also served as habituation for the novel position test on the next day. The behavior from the first 5 mins were analyzed to evaluate the anxiety level and the behavior from the first 30 mins were analyzed to assess the locomotor activity.

***Novel position test.*** Two days after the open field test, mice were released into the same arena with visual cues on the arena walls. Two identical silicone vials were placed in the area as shown in **Fig. 2f**. Mice were allowed to interact with vials for 30 mins and returned to the home cage. One hr after training, one of the silicone vials was moved to a new location and mice were placed back to the arena for 10 mins. The mouse's position was continuously recorded by Limelight 4. The location of the novel position was counterbalanced during tests. The objects were thoroughly cleaned with Quatricide between tests to minimize olfactory cues. During offline analysis after the experiment, a two-cm virtual band around the silicone vial was drawn in Limelight 4 and the nose position within the band was tracked to reflect the exploration. Data are presented as a

discrimination index, which is the difference of the amount of interaction time (novel position – familiar position) divided by the total interaction time (novel position + familiar position) (6).

***Barnes maze test.*** The circular maze table was 92 cm in diameter, 105 cm in height with 20 equally spaced holes (5 cm diameter) around the perimeter (7, 8). The maze was positioned at the center of a square area (1.7 x 1.7 $m^2$) surrounded by curtains and each side of the curtain was decorated with visual cues. Two bright lights were turned on during testing (~1500 lux at the maze level). During training, 19 of the holes were blocked and one hole led to an escape box (target hole). Each mouse was released in the middle of the maze by removing a cylindrical black start tube and allowed to explore the maze for 3 mins. If the mouse entered the target hole, it was returned back to its home cage after 1 min. If the mouse failed to find the target hole, it was gently guided to the target hole and allowed to stay in the escape box for 1 min. Fifteen mins later, the mouse was trained again, and such training was repeated for a total of 4 times each day for 4 consecutive days. The target hole for each mouse was randomized. The total latency to the target hole was recorded when all four paws of the mouse were inside the escape box. On the fifth and twelfth day, probe trials were run where all the holes were blocked and the amount of time that mouse spent in the virtual target hole area within 2 mins was measured. The maze was cleaned with diluted *Quatricide* and dried between each trial to remove olfactory cues. Mouse behavior was recorded with Limelight 4.

## Genotyping and Cloning

***$Kcnc3^{Clue/Clue}$ mutation genotyping.*** DNA region containing the *Clueless* mutation was amplified and sequenced with following primers.
Amplifying primers – 836 bp amplicon

Forward primer 5’-GGCCACCACCAAGTTCTTTA-3’

Reverse primer 5’-GCCGAAGTTGTTGACAATGA-3’

Sequencing primer 5’-CAAAAGCAGCCTGAACATCA-3’

Allele specific PCR primers

Forward primer for WT allele 5’-CTGACCCGTCACTTCGTAGG-3’

Forward primer for *Clueless* allele 5’-CTGACCCGTCACTTCGAGGT-3’

Reverse primer 5’-ATGGTGGCAAAGATGAGGAC-3’

Real-time PCR primers

Forward primer 5’-CTGACCCGTCACTTCGTGGG-3’

Reverse primer 5’-ATGGTGGCAAAGATGAGGAC-3’

Regular PCR was performed with KAPA2G fast HS readyMix (KM5610, KAPA, Wilmington, MA) following manufacturer's instructions. Thermo-cycling reaction conditions were as follows: 1 cycle of 98 °C (5 min), 35 cycles of 98 °C (15 sec), 58 °C (15 sec) and 72 °C (1 sec), ended with 1 cycle of 72 °C (2 min). Real-time PCR was performed with 2x SYBR supermix (#1725275, BioRad, Hercules, CA) following manufacturer's instructions. PCR conditions were 95 °C (3 min), 40 cycles of 95 °C (3 sec) and 64 °C (20 sec). Data was collected with an ABI 7900HT Fast Real-Time PCR System (Applied Biosystems, Inc.).

***Kcnc3 cloning and site-directed mutagenesis.*** RNA from WT C57Bl/6J hippocampus was isolated with Direct-zol RNA MiniPrep Kit (R2050, Zymo Research, Tustin, CA). cDNA was synthesized with Taqman Reverse Transcription kit from Applied Biosystems (Thermofisher, Foster City, CA) following manufacturer's instructions. *Kcnc3* cDNA (variant 1, uc009gqg.1) was cloned with the following primers into pcDNA3.1 vector (Thermofisher, Foster City, CA).

Forward primer 5'-CGGCAAGCTTGCCACCATGCTCAGTTCAGTGTGCGTCT-3'

Reverse primer 5'-GCGCGGATCCAGGGCTGCGCTAGAGGAT-3'

*Kcnc3*$^{G434V}$ and *Kcnc3*$^{(1\text{-}465)}$ was created by site direct mutagenesis with Platinum Pfx DNAPolymerase (Thermofisher, Foster City, CA) following manufacturer's instructions. In brief, PCR conditions were 94 °C (5 min), 15 cycles of 94 °C (30 s), 50 °C (30 s) and 72 °C (15 min). PCR products were digested with DpnI (New England Biolabs, Ipswich, MA) overnight at 37 °C and transformed into *E. coli*. Correct plasmids were confirmed with sequencing.

*Kcnc3*$^{G434V}$_forward primer

5'-CTGACCCGTCACTTCGTGGTGCTGCGTGTGCTGGGCCAC-3'

*Kcnc3*$^{G434V}$_reverse primer

5'-GTGGCCCAGCACACGCAGCACCACGAAGTGACGGGTCAG-3'

*Kcnc3*$^{(1\text{-}465)}$_forward primer

5'-CAAGCTGACCCGTCACTTGTGGGGCTGCGTGTGCTG-3'

*Kcnc3*$^{(1\text{-}465)}$_reverse primer

5'-CAGCACACGCAGCCCCACAAGTGACGGGTCAGCTTG-3'

For FLAG tagged KCNC3, KCNC3 (G434V) and KCNC3 (1-465), the corresponding DNA sequences were excised from pcDNA3.1 vector and inserted into p3XFLAG-CMV-9 Expression Vector (E9783, Sigma).

## Sequencing and Gene Expression Analysis

***Stranded mRNA-seq.*** Raw RNA-seq data (fastq files) were trimmed based on quality score and N calling. For quality score, reads showing poor quality scores (< 20) from both 5'-end 10bp and 3'-end 38bp windows were trimmed. For N calling, reads containing Ns at 3'-end 15bp window were trimmed. Short trimmed reads (< 30 bp) and reads with poor average quality scores (< 21) or low read accuracy values (< -1) were also trimmed. From the read accuracy value cut off, trimmed reads could be filtered if they had < 50% probability to be accurate. Read accuracy values were calculated using the following formula, where k is the length of a trimmed read:

$$Read\ accuracy\ value = \sum_{n=1}^{k} \log_2(1 - 10^{-\frac{quality\ score_n}{10}})$$

After the quality score and N calling trimming, reads were further trimmed to remove contamination of adapters and polyA signals using cutadapt (version 1.14) (9) using the following parameters:

Adapter trimming: cutadapt -g AATGATACGGCGACCACCGAGATCTACACTCTTTCCCTACACGACGCTCTTCCGATCT -a AGATCGGAAGAGCACACGTCTGAACTCCAGTCAC -m 30

PolyA signal trimming: cutadapt -g T{100} -m 30

The sequence qualities of fastq files were tested before and after the trimming process using both FastQC (version 0.11.8) (Babraham Bioinformatics - FastQC A Quality Control tool for High Throughput Sequence Data: https://www.bioinformatics.babraham.ac.uk/projects/fastqc/) and FastQ Screen (version 0.13.0) (10).

After read trimming, a STAR index was prepared using the M14 reference genome (GRCm38.p5), only considering chromosomes without contigs using a matched gtf file (gencode.vM14.annotation.gtf.gz). The trimmed fastq reads were aligned to the index using STAR (version 2.5.3a) (11) with the following parameters:

STAR --runMode alignReads --alignSJoverhangMin 10 --alignSJDBoverhangMin 1 --alignIntronMax 1000000 --outFilterMultimapNmax 10 --outFilterMismatchNmax 3

From uniquely mapped reads, reads from 45,141 genes containing transcripts (≧ 200bp) were considered read alignment directions using featureCounts (version 1.6.0) (12) using the following parameters:

```
featureCounts -s 2 -O -t exon -g gene_id
```

From read counts aligned to the forward direction of transcripts, RPKM values were calculated after adding one more count to avoid missing value errors during $\log_2$ transformation (13).

***Differential gene expression analysis.*** Using bam files from STAR alignment, summary statistics for quality control were generated from: CollectAlignmentSummaryMetrics, CollectRnaSeqMetrics, CollectGcBiasMetrics, and MarkDuplicates arguments of Picard version 2.19.1 (http://broadinstitute.github.io/picard/). Metadata were generated including the summary statistcs (**Dataset S4**).

Lowly expressed genes in the hippocampus were filtered out using uniquely mapped read counts and mean read ratio (read ratio = read count /(transcript length(bp) / 3,000)). Genes were filtered if both measures were < 3. To overcome variation in mapped read numbers across samples, genes were further filtered if the read counts and read ratios of sample with minimum mapped reads were < 2. Therefore,16,009 expressed genes (35.464%) out of 45,141 genes (transcript length ≧ 200bp) remained after filtering. The distribution of $\log_2$(RPKM) was close to a normal distribution without showing noise-level expressed genes (**Fig. S10**).

Mapped read data were normalized to decrease variance introduced by uncontrolled influences. A normalized RPKM was calculated, considering both gene length and GC content, from read count using CQN (conditional quantile normalization) R package (14). MA plots showed that the normalized RPKM values generate the expected MA plots and are unlikely to be the previously calculated RPKM values (**Fig. S10**). Though the MA plot of the previously calculated RPKM values showed mostly positive log(fold change) ($M > 0$) in high expressed genes (e.g. $A > 10$), the pattern disappeared at the MA plot of the normalized RPKM values.

Outlier samples were removed if their Z score of overall connectivities across all genes was > 2. Connectivity is a measure of how correlated a gene is with all other genes in the dataset. Outlier samples also tend to have a large clustering coefficient. The clustering coefficient show how gene clusters are intraconnected, and high clustering coefficient values imply that all genes are connected with each other. Therefore, high clustering coefficient and low connectivity implies little replication of a true biological system in the samples. Outlier samples with low connectivities were assessed for high clustering coefficients (**Fig. S7**), leading to the filtering of two additional outlier samples (C05_2_MT_Ctl_DDG and C13_3_MT_FCT_DDG; see **Dataset S4** for detailed information).

From principal component analysis of the metadata, allowed for identification first and second principal components containing sequence statistics contributed by the metadata components that influence gene expression. From the principal components and metadata components, one-way anova tests were performed across all samples to find confounding factors. Metadata components were considered as independent compounding factors if they showed significant p-values from the anova as well as poor correlations with the first and second principal compoents. Using limma R package, the influences of independent confounding factors were regressed out from gene expression data using the linear model below:

$\log_2$(normalized RPKM) ~ Age + RIN + PF_HQ_ERROR_RATE + INTERGENIC_BASES + AT_DROPOUT + PC1 + PC2

Wherein $\log_2$(normalized RPKM) is the gene expression data, and RIN is RNA integrity numbers. PF_HQ_ERROR_RATE, INTERGENIC_BASES, and AT_DROPOUT are from the summary statistics from Picard (http://broadinstitute.github.io/picard/). PC1 and PC2 are the first and second principal components. From the gene expression data, 32 differential gene expression analyses were performed for control vs FCT, wild type vs *Clueless*, dorsal vs ventral, and DG vs CA (**Dataset S5**). DEGs were identified if their p-values were below 0.01.

a

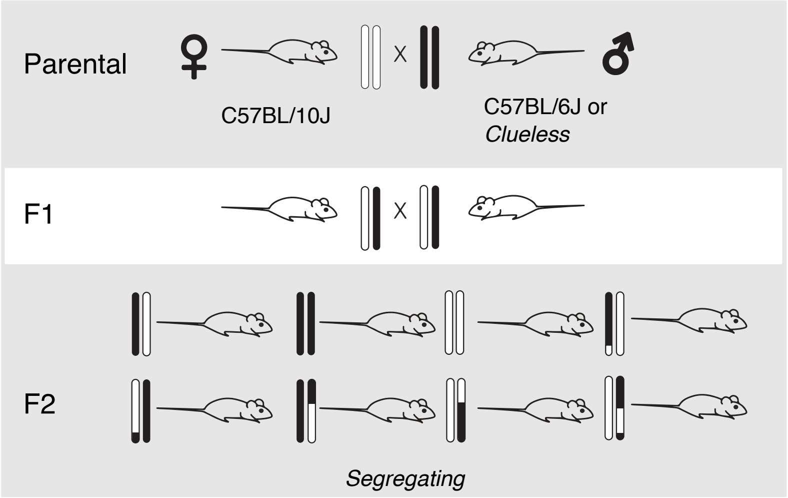


b

$$h^2 = \frac{V_{Genetic}}{V_{Phenotype}} = \frac{\sigma^2_{genetic}}{\sigma^2_{genetic} + \sigma^2_{environment}}$$

$$\sigma^2_{environment} = \frac{\sigma^2_{B6J} + \sigma^2_{B10J} + \sigma^2_{F1}}{3}$$

$$\sigma^2_{F2} = \sigma^2_{genetic} + \sigma^2_{environment}$$

$$\sigma^2_{genetic} = \sigma^2_{F2} - \sigma^2_{environmnet}$$

$$h^2 = 11.22\ \%$$

$$\frac{\text{Fitqtl estimated variance}}{\text{Total observed variance}} = \frac{10.75\ \%}{11.22\ \%} = 95.8\ \%$$

**Figure S1: Breeding scheme for QTL mapping and heritability analysis. a)** Wild type C57BL/10J female mice were mated with wild type C57BL/6J male mice to produce F1 mice (WTB10B6F1). WTB10B6F1 mice were intercrossed to produce ~ 250 F2 mice (WTB10B6F2) for mapping potential QTLs that contributed to differences in contextual fear conditioning phenotype between C57BL/10J and C57BL/6J. Similarly, to map the causative locus in *Clueless* mutants, wild type C57BL/10J female mice were mated with *Clueless* male mice (B6J background) to produce F1. F1 mice with low contextual freezing scores were intercrossed to produce ~ 250 F2 mice for mapping. All mice were tested concurrently. **b)** B6J, B10J and WTB10B6F1 are three isogenic lines. We took the mean of the 3 variances to represent the environmental variance. The variance from *Clue*B10B6F2 was caused by both genetic and environmental factors. Based on the equation and the SD from **Fig. 1e**, the heritability was

calculated to be ~ 11.2%. Fitqtl function in QTL/R suggests that the QTL on chr7 contributes to 10.75% total phenotypic variance. Therefore, this QTL explains 96% (10.75/11.2) of genetic variance.

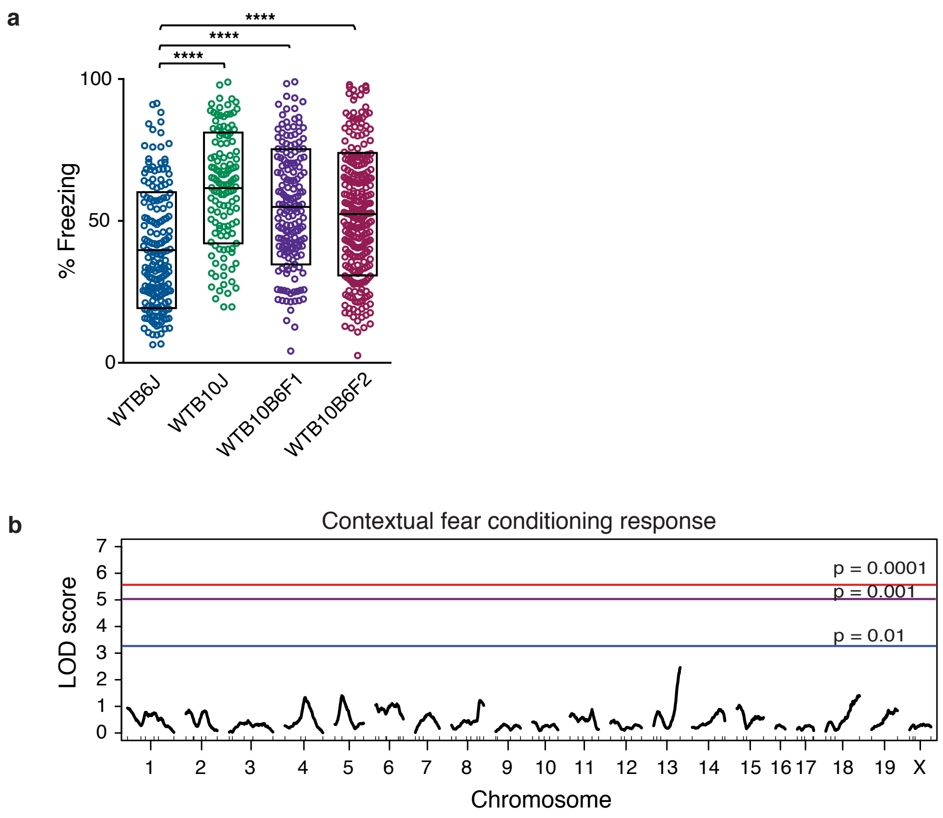


**Figure S2: QTL analysis in WTB10B6F2. a)** % freezing time in WTB6J ($n$ = 178), WTB10J ($n$ = 128), WTB10B6F1 ($n$ = 174) and WTB10B6F2 mice ($n$ = 264). Mean ± s.d. are shown. (**** $P < 0.001$, one-way ANOVA followed by Tukey's test, $F_{3,740} = 31.39$, ****$P < 0.0001$). **b)** Genome-wide QTL scan for contextual fear conditioning response in WTB10B6F2. The significance thresholds were calculated with 10,000 permutation tests.

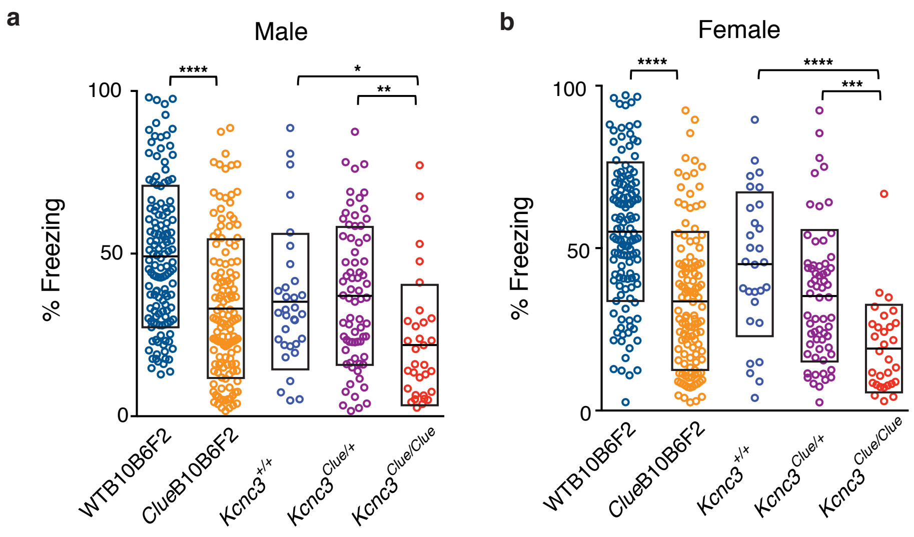


**Figure S3: Fear conditioning in male and female *Clueless* mice in B10B6 background. a)** Males, WTB10B6F2 ($n$ = 136), *Clue*B10B6F2 ($n$ = 137), *Kcnc3*$^{+/+}$ ($n$ = 32), *Kcnc3*$^{Clue/+}$ ($n$ = 73), *Kcnc3*$^{Clue/Clue}$ ($n$ = 32). (**** $P$ <0.0001, * $P$ < 0.05, Kolmogorov-Smirnov test was used for two group comparison and one-way ANOVA followed by Tukey's was used for among genotype analysis, $F_{2,134}$ = 6.258, **$P$ = 0.0025). **b)** Females, WTB10B6F2 ($n$ = 131), *Clue*B10B6F2 ($n$ = 122), *Kcnc3*$^{+/+}$ ($n$ = 29), *Kcnc3*$^{Clue/+}$ ($n$ = 63), *Kcnc3*$^{Clue/Clue}$ ($n$ = 30). Mean ± s.d. are shown. (**** $P$ <0.0001, Kolmogorov-Smirnov test was used for two group comparison and one-way ANOVA followed by Tukey's test was used for among genotype analysis, $F_{2,119}$ = 13.78, ****$P$ < 0.0001).

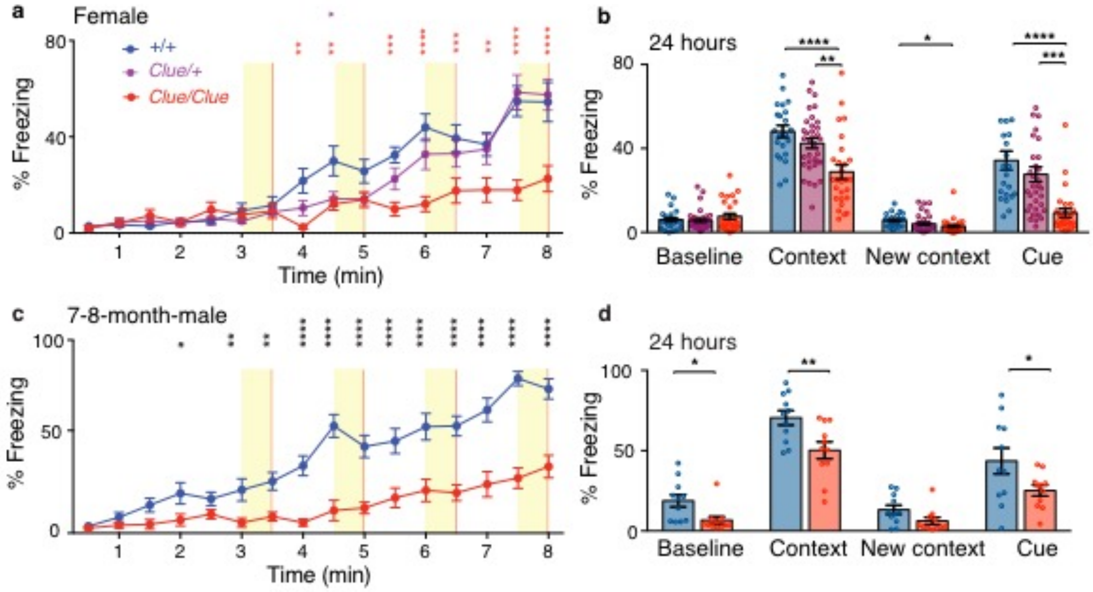


**Figure S4: Fear conditioning phenotypes in female and 7-8-month-old male *Clueless* mutants. a)** Female *Clueless* mutants showed reduced freezing timing during training. (*+/+*, *n* = 17; *Clue/+,* *n* = 18; *Clue/Clue*, *n* = 18. **** *P* <0.0001, *** *P* <0.001, ** *P* <0.01, * *P* <0.05, two-way repeated measures ANOVA, interaction genotype X time, $F_{30, 750}$ = 6.178, ****$P$ < 0.0001; time effect, $F_{15, 750}$ = 81.63, ****$P$ < 0.0001, genotype effect, $F_{2, 50}$ = 9.283, ****$P$ <0.0004, adjusted with Tukey's post hoc test). **b)** Female *Clueless* mutants showed reduced contextual fear condition responses 24 hrs after training. (*+/+*, *n* = 22; *Clue/+,* *n* = 34; *Clue/Clue*, *n* = 27. **** *P* <0.0001, One-way ANOVA adjusted with Tukey's test, contextual % freezing, $F_{2, 80}$ = 11.29, $P$ < 0.0001; cued % freezing, $F_{2, 80}$ = 12.76, ****$P$ < 0.0001; baseline % freezing, $F_{2, 80}$ = 0.8831, $P$ = 0.4175; changed context baseline % freezing, $F_{2, 80}$ = 3.587, * $P$ = 0.0322). **c)** Older male *Clueless* mutants showed reduced freezing timing during training session. (*+/+*, *n* = 11; *Clue/Clue*, *n* = 11. **** *P* <0.0001, *** *P* <0.001, ** *P* <0.01, * *P* <0.05, two-way repeated measures ANOVA: interaction genotype X time, $F_{15, 150}$ = 11.37, ****$P$ <0.0001; genotype effect, $F_{1, 10}$ = 69.07, ****p <0.0001; time effect, $F_{15, 150}$ = 30.97, ****p <0.0001, adjusted with Sidak's post hoc test). **d)** Older male *Clueless* mutants showed reduced contextual fear conditioning responses 24 hrs after training. (*+/+*, *n* = 11; *Clue/Clue*, *n* = 11. ** *P* < 0.01, * *P* < 0.05. Unpaired student *t* test). Mean ± s.e.m. are shown.

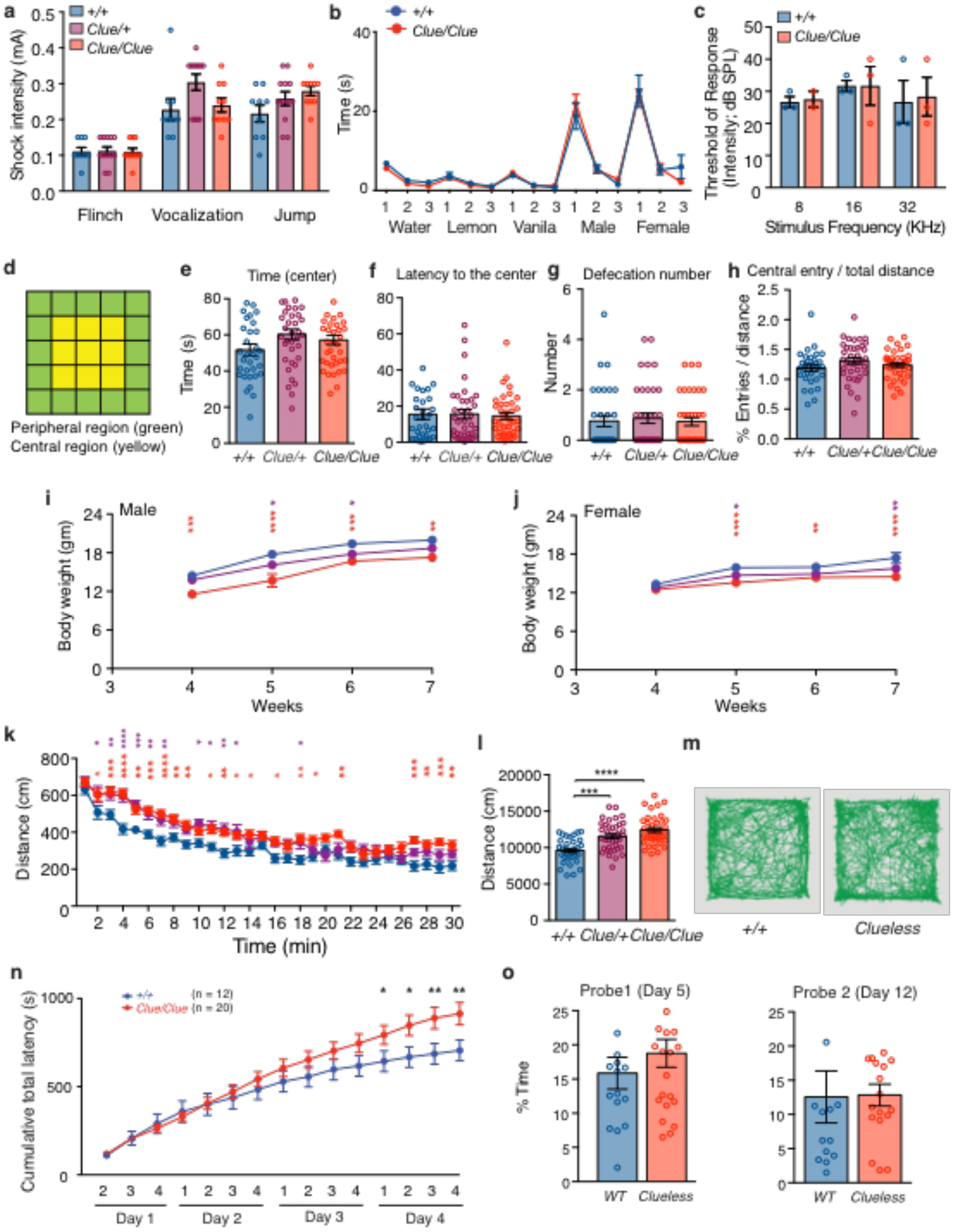


**Figure S5: *Clueless* mutants demonstrate normal foot shock, olfactory habituation responses, normal anxiety-like responses, reduced body weight and elevated locomotor activity. a)** Foot shock responses in male littermates (*+/+*, *n* = 9; *Clue/+, n* = 12; *Clue/Clue, n* = 10). (Tukey's post hoc test following one-way ANOVA. Flinch, $F_{2, 28}$ = 0.01441, $P$ = 0.9857; vocalization, $F_{2, 28}$ = 3.08, $P$ = 0.0618; jump, $F_{2, 28}$ = 2.597, $P$ = 0.0923). **b)** Olfactory habituation

responses in male littermates *+/+* (*n* = 9) and *Clue/Clue* (*n* = 10). (Sidak's post hoc test following two-way repeated measures ANOVA: interaction genotype x time, $F_{14, 238} = 0.7743$, $P = 0.6966$; genotype effect, $F_{1, 17} = 0.1123$, $P = 0.7417$; time effect, $F_{14, 238} = 67.97$, ****$P < 0.0001$). **c)** Hearing sensitivity test in male littermates *+/+* (*n* = 3) and *Clue/Clue* (*n* = 3). (Unpaired t test, 8 KHz, $P = 0.7888$; 16 KHz, $P > 0.9999$; 32 KHz, $P = 0.8617$). **d)** Schematic of the matrix used in the assay (peripheral region is showed in green and center is showed in yellow). **e)** time spent in the center, **f)** latency of first entry to the center, **g)** number of fecal boli collected. **h)** number of center entries normalized with the total distance traveled. (*+/+*, *n* = 33; *Clue/+, n* = 38; *Clue/Clue, n* = 38. Tukey's post hoc test following one-way ANOVA. No difference was seen among genotypes). The body weights of male **(i)** and female **(j)** mice were measured weekly from the age of 4 weeks to 7 weeks. (Male, *+/+*, *n* = 11-15; *Clue/+*, *n* = 15; *Clue/Clue, n* = 11-14. **** $P$ <0.0001, *** $P$ <0.001, ** $P$ <0.01, * $P$ <0.05, two-way ANOVA, interaction genotype X time, $F_{6, 157} = 0.7294$, $P = 0.6266$; time effect, $F_{3, 157} = 71.88$, **** $P$ <0.0001, genotype effect, $F_{2, 157} = 37.62$, **** $P$ <0.0001; Female, *+/+*, *n* = 7-18; *Clue/+, n* = 11-18; *Clue/Clue*, *n* = 14-18, interaction genotype x time, $F_{6, 174} = 1.233$, $P = 0.2916$; time effect, $F_{3, 174} = 26.27$, ****$P$ <0.0001, genotype effect, $F_{2, 174} = 23.58$, **** $P$ <0.0001, adjusted with Tukey's post hoc test). **k)** The distance traveled per min over 30 mins. *Clueless* mice habituated to the open field more slowly than WT mice and as a consequence showed longer travel distance than WT. The slower habituation rate is consistent with the learning deficit in *Clueless* mice. (*+/+*, *n* = 26; *Clue/+, n* = 18; *Clue/Clue*, *n* = 26. **** $P$ <0.0001, *** $P$ <0.001, ** $P$ <0.01, * $P$ <0.05, two-way repeated measures ANOVA: interaction genotype x time, $F_{58, 1943} = 1.309$, $P = 0.0609$; time effect, $F_{29, 1943} = 60.2$, **** $P$ <0.0001, genotype effect, $F_{2, 67} = 19.14$, **** $P$ <0.0001; adjusted with Tukey's post hoc test). **l)** *Clueless* showed increased average traveled distance over 30 mins. (*+/+*, *n* = 33; *Clue/+, n* = 38; *Clue/Clue, n* = 38. **** $P$ <0.0001, *** $P$ <0.001, one-way ANOVA, adjusted with Tukey's post hoc test, $F_{2, 106} = 19.85$, **** $P$ <0.0001). **m)** Sample tracking data from *WT* and *Clueless* mice. **n)** Accumulated latency for mice to locate the target hole were plotted during training trials for WT (blue, *n* =12) and *Clueless* littermates (red, *n* = 20). (** $P$ <0.01, * $P$ <0.05, Fisher's LSD test following two-way repeated measures ANOVA, interaction genotype X time, $F_{14, 420} = 5.271$, $P < 0.0001$; time effect, $F_{14, 420} = 153.7$, $P$ <0.0001; genotype effect, $F_{1, 30} = 1.564$, $P = 0.2207$). **o)** Time spent around the virtual target hole region 1 day (day 5, $P = 0.3572$) and 7 days (day 12, $P = 0.943$) after training. Unpaired *t* test. Mean ± s.e.m. are shown.

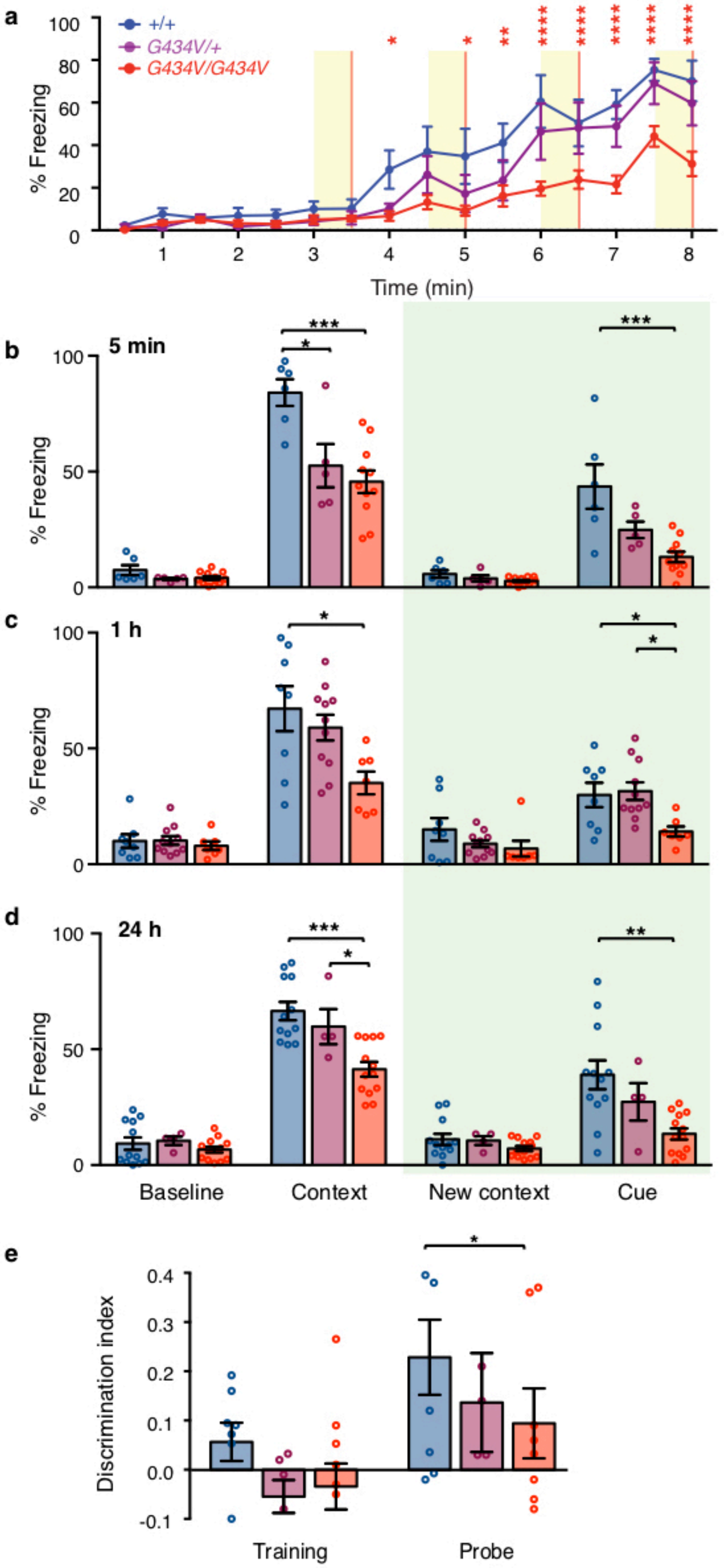
a
+/+
G434V/+
G434V/G434V
% Freezing
Time (min)
b
5 min
c
1 h
d
24 h
Baseline
Context
New context
Cue
e
Discrimination index
Training
Probe

**Figure S6**: **CRISPR/Cas9 engineered *Kcnc3*$^{G434V/G434V}$ mice show deficits in both fear conditioning and novel position tests.** Data from Kcnc3_L50; **a)** % of freezing time during training. (*+/+, n* = 12; *Kcnc3*$^{G434V/+}$, *n* = 5; *Kcnc3*$^{G434V/G434V}$, *n* = 17. ****$P$ <0.0001, ** $P$ <0.01, two-way repeated measures ANOVA, interactions genotype X time, $F_{30, 465}$ = 5.208, ****$P$ <0.0001; genotype effect, $F_{2, 31}$ = 9.727, *** $P$ = 0.0005; time effect, $F_{15, 465}$ = 65.11, ****, $P$ <0.0001; adjusted with Tukey's post hoc test). **b)** % of freezing time 5 min after training (*+/+, n* = 6; *Kcnc3*$^{G434V/+}$, *n* = 5; *Kcnc3*$^{G434V/G434V}$, *n* = 11. *** $P$ <0.001, * $P$ <0.05, one-way ANOVA, adjusted with Tukey's post hoc test, contextual % freezing, $F_{2, 19}$ = 10.49, ***$P$ < 0.0009; cued % freezing, $F_{2, 19}$ = 9.546, **$P$ = 0.0013; baseline % freezing, $F_{2, 19}$ = 2.403, $P$ = 0.1174; changed context baseline % freezing, $F_{2, 19}$ = 2.262, $P$ = 0.1315). **c)** % freezing time tested 1 hr after training (+/+, *n* = 8; *Kcnc3*$^{G434V/+}$, *n* = 11; *Kcnc3*$^{G434V/G434V}$, *n* = 7. * $P$ <0.05, one-way ANOVA, adjusted with Tukey's post hoc test, contextual % freezing, $F_{2, 23}$ = 4.907, *$P$ = 0.0168; cued % freezing, $F_{2, 23}$ = 4.946, *$P$ = 0.0163; baseline % freezing, $F_{2, 23}$ = 0.2787, $P$ = 0.7593; changed context baseline % freezing, $F_{2, 23}$ = 1.598, $P$ = 0.2239). **d)** % freezing time tested 24 hrs after training (+/+, *n* = 12; *Kcnc3*$^{G434V/+}$, *n* = 4; *Kcnc3*$^{G434V/G434V}$, *n* = 13. *** $P$ <0.001, ** $P$ <0.01, one-way ANOVA, adjusted with Tukey's post hoc test, contextual % freezing, $F_{2, 26}$ = 12.3, ***$P$ = 0.0002; cued % freezing, $F_{2, 26}$ = 7.78, **$P$ = 0.0022; baseline % freezing, $F_{2, 26}$ = 0.6585, $P$ = 0.5260, changed context baseline % freezing, $F_{2, 26}$ = 0.877, $P$ = 0.4280). **e)** Novel position test, *+/+* (*n* = 8), *Kcnc3*$^{G434V/+}$ (*n* = 6), *Kcnc3*$^{G434V/G434V}$ (*n* = 10). One sample *t* test, two tailed. Training, *+/+*, $P$ = 0.1085, *Kcnc3*$^{G434V/+}$, $P$ = 0.1626, *Kcnc3*$^{G434V/G434V}$, $P$ = 0.4826. Test at 1 hr, *+/+*, *$P$ = 0.0201, *Kcnc3*$^{G434V/+}$, $P$ = 0.2325, *Kcnc3*$^{G434V/G434V}$, $P$ = 0.2188. Mean ± s.e.m. are shown.

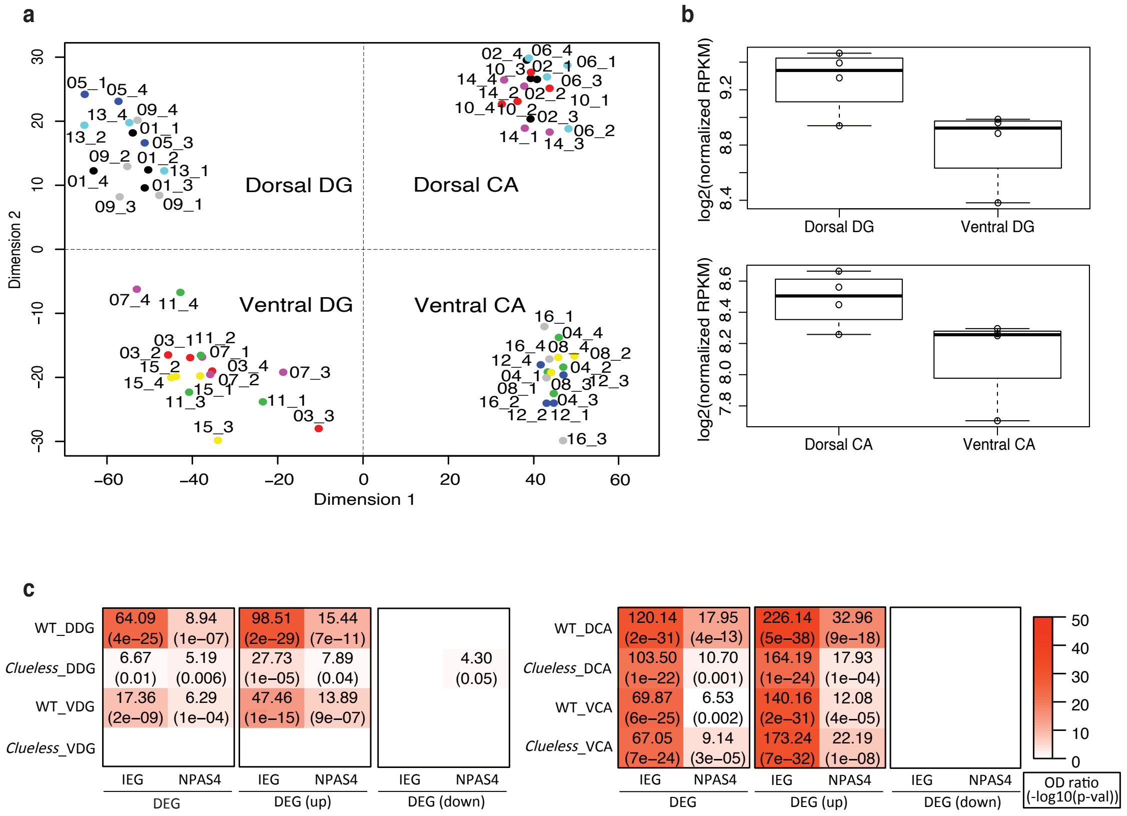


**Figure S7: Differential Gene Expression Analysis in the Hippocampus of WT and *Clueless* mice. a)** Multidimensional scaling plot generated by $\log_2$ (normalized RPKM) values of stranded mRNA-seq data from WT control and fear conditioned mice. The datapoints represent individual RNA-seq samples from 4 different regions of the hippocampus. The numbers next to the dots represent the Sample_ID found in column 2 of **Dataset S4**. Hippocampal region played a larger role in changing gene expression than fear conditioning. **b)** Boxplots showing *Kcnc3* expression in the dorsal and ventral hippocampus of WT control mice (n= 4/region). The one-tailed t-test p-values are 0.02265 and 0.04295 in DG (top) and CA (bottom), respectively. **c)** Gene set enrichment analysis comparing immediate early genes (IEGs) and *Npas4* target genes (*Npas4*) in response to fear conditioning in WT and *Clueless* mice. WT mice had more IEGs and NPAS4 target genes with differential expression after fear conditioning than *Clueless* mice.  DDG = dorsal dentate gyrus, VDG = ventral dentate gyrus, DCA = dorsal cornu ammonis, and VCA = ventral cornu ammonis.  Darker red boxes indicate more genes with changes in expression.

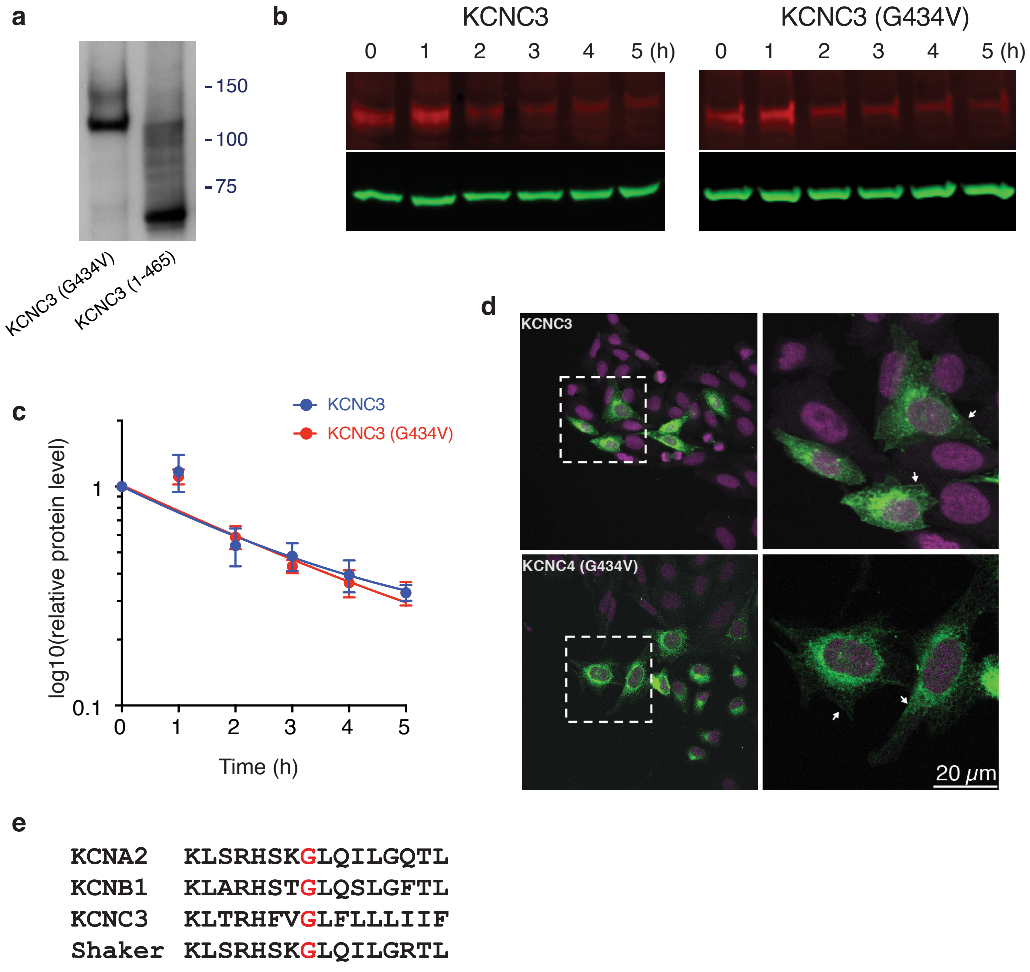


**Figure S8: KCNC3(1-465) produces a truncated protein in transfected CHO cells and KCNC3(G434V) has a similar half-life as KCNC3 in transfected CHO cells**. Sanger sequencing showed that KCNC3$^{1\text{-}465}$ mice had a cytosine missing at chr7: 44,595,583 (mm10), which resulted in a frame shift creating a nearby stop codon. The C-terminally truncated protein was predicted to have only 465 amino acids. To verify this, we recreated the loss of cytosine in a construct containing the full length KCNC3 cDNA by point mutagenesis. **a)** Both *Kcnc3*$^{1\text{-}465}$ and *Kcnc3*$^{G434V}$ constructs had a FLAG tag attached to its N-terminus and were transfected into CHO cells. Western blot analysis demonstrated that the full-length FLAG- KCNC3$^{G434V}$ was ~110 kDa whereas the FLAG-KCNC (31-465) was ~51 kDa (the predicted size). **b)** Protein half-life was analyzed by cycloheximide treatment in transfected CHO cells. Representative immunoblots of KCNC3 (red) and ß-actin (green) after 0, 1, 2, 3, 4 and 5 hrs of 20 μg/ml cycloheximide treatments. **c)** The half-life was calculated by nonlinear one phase decay method. Nonlinear fitting curves were shown on log10 scale. KCNC3$^{WT}$ and KCNC3$^{G434V}$ have similar half-life. (KCNC3, $T_{1/2}$= 1.9 h, KCNC3 (G434V),

$T_{1/2}$=2.3 h). Mean ± s.e.m. are shown. **d)** Expression of $KCNC3^{WT}$ and $KCNC3^{G434V}$ in transfected CHO cells. CHO cells transiently co-transfected with either $KCNC3^{WT}$ or $KCNC3^{G434V}$ along with an eGFP expressing construct were stained with an KCNC3 antibody (Green). TOPRP3 staining (purple) labeled the nucleus. Higher magnification images (white square) were on the right. Scale bar, 20 $\mu$m. Arrows indicate membrane staining. **e)** Sequence alignment demonstrated that Glycine 434 is conserved among different members of voltage dependent potassium channels in mice.

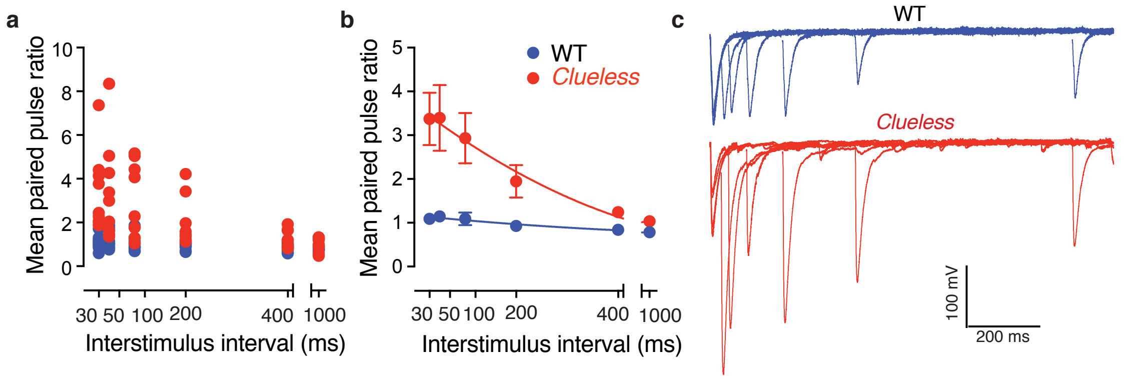


**Figure S9: Paired-pulse ratio at MF-CA3 synapses is enhanced in *Clueless* mice. a)** Individual paired-pulse ratio values in WT ($n$ = 9 cells, 3 mice) and *Clueless* mice ($n$ = 9 cells, 3 mice) are shown for different interstimulus intervals. **b)** Mean paired-pulse ratio obtained from values shown in (**a**). (Two-way repeated measures ANOVA, interaction genotype X interstimulus interval, $F_{5, 80}$ = 5.835, ***$P$ = 0.0001; genotype effect, $F_{1, 16}$ = 14.04, ** $P$ = 0.0018). **c)** Superimposed averaged sample traces from WT and *Clueless* at different interstimulus intervals. Traces have had stimulus artifacts removed and are averages of 8-10 consecutive responses. The first averaged EPSC was obtained from the first stimulation, and then followed by averaged EPSCs obtained at 30, 50, 100, 200, 400, or 1000 ms interstimulus interval. Mean ± s.e.m. are shown.

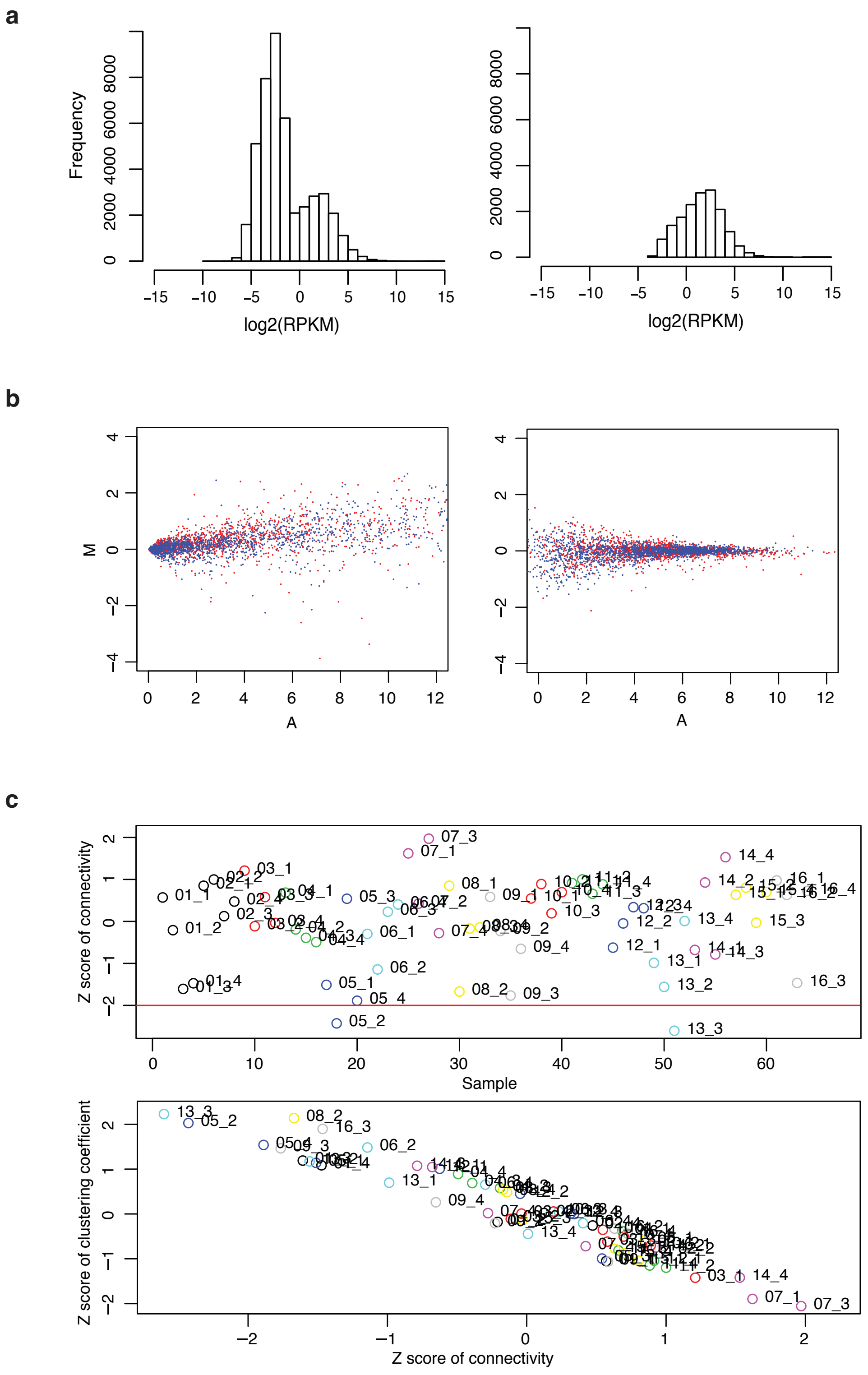
a
Frequency
log2(RPKM)
log2(RPKM)
b
M
A
A
c
Z score of connectivity
Sample
Z score of clustering coefficient
Z score of connectivity

**Figure S10**: **Differential gene expression analysis**. a) Histograms of $log_2$(RPKM) values before (left) and after (right) filtering low-expression genes. b) MA plots after CQN normalization. For the MA plots, two groups of stranded mRNA-seq samples (4 samples of C01_#_WT_Ctl_DDG and 4 samples of C09_#_WT_FCT_DDG; #s are Replicate_num in **Dataset S4**). Before CQN normalization, RPKM values were used for a MA plot (left). The other MA plot (right) was generated with normalized RPKM values. The red dots represent genes with high 10% GC ratios, and the blue dots represent genes with low 10% GC ratios. c) Plots for overall connectivity and clustering coefficient to remove outlier samples. Z scores of connectivity were ploted for all stranded mRNA-seq samples (top). The red line indicates -2 of the Z score. The Z scores of connectivity and Z scores of clustering coefficient were ploted for all samples (bottom).

**Dataset S1: SNP panel used for QTL mapping**

**Dataset S2: GATK analysis**

**Dataset S3: Gene expression comparisons for all known K channels between WT and *Clueless* mice.** For all known K channels, expression is shown in $log_2$(normalized RPKM). The second row shows considered sample names, from the Sample_name in **Dataset S4**. Groups were compared to identify expression difference between WT and *Clueless* mice. Comparisons are listed on the second row, and the comparison list is from **Dataset S5**. Log2(fold change) and p-values are shown for each comparison.

**Dataset S4:** Metadata of strand mRNA-seq data. Information on samples, strand mRNA-seq libraries, statistics of fastq read trimming and read mapping, and summary statistics for QC generated by Picard. The column titles of the summary statistics are capitalized.

**Dataset S5:** Gene expression analysis output. The first sheet contains overall information of all gene expression analyses. Analysis_name column has the names of all gene expression analyses between two sample groups (Group_1 and Group_2). The names became the titles of the other 32 sheets. The sample names of Group_1 and Group_2 columns are from Sample_name in **Dataset S4**. #s in the sample names indicate Replicate_num are from **Dataset S4**. If there were no filtered outliers, all replicates were used for gene expression analyses.

**Dataset S6:** Gene lists for gene set analysis including all DEGs. The first sheet, IEG_Npas4, contains IEG and Npas4 target genes. DEG sheet contains all lists of DEGs from 32 gene expression analyses. Column names match the Analysis_name column in **Dataset S5**. DEG_up and DEG_down sheets show the subsets of DEGs that show up- or down-regulated DEGs, respectively. Background_gene sheet shows the background gene list that used for gene set enrichments, which was a list of all (16,009 genes) expressed in the mouse hippocampus.

**SI References**


1. M. S. Fanselow, Factors governing one-trial contextual conditioning. *Animal Learning & Behavior* **18**, 264–270 (1990).
2. R. Paylor, R. Tracy, J. Wehner, J. W. Rudy, DBA/2 and C57BL/6 mice differ in contextual fear but not auditory fear conditioning. *Behav Neurosci* **108**, 810-817 (1994).
3. J. M. Wehner, R. A. Radcliffe, Cued and contextual fear conditioning in mice. *Curr Protoc Neurosci* **Chapter 8**, Unit 8 5C (2004).
4. M. Yang, J. N. Crawley, Simple behavioral assessment of mouse olfaction. *Curr Protoc Neurosci* **Chapter 8**, Unit 8 24 (2009).
5. V. Kumar *et al.*, C57BL/6N mutation in cytoplasmic FMRP interacting protein 2 regulates cocaine response. *Science* **342**, 1508-1512 (2013).
6. T. Murai, S. Okuda, T. Tanaka, H. Ohta, Characteristics of object location memory in mice: Behavioral and pharmacological studies. *Physiol Behav* **90**, 116-124 (2007).
7. S. P. Berta Sunyer , Harald Höger, Gert Lubec Barnes maze, a useful task to assess spatial reference memory in the mice *Protocol Exchange* 10.1038/nprot.2007.390 (2007).
8. F. E. Harrison, A. H. Hosseini, M. P. McDonald, Endogenous anxiety and stress responses in water maze and Barnes maze spatial memory tasks. *Behav Brain Res* **198**, 247-251 (2009).
9. M. Martin, Cutadapt removes adapter sequences from high-throughput sequencing reads. *2011* **17**, 3 (2011).
10. S. W. Wingett, S. Andrews, FastQ Screen: A tool for multi-genome mapping and quality control. *F1000Res* **7**, 1338 (2018).
11. A. Dobin *et al.*, STAR: ultrafast universal RNA-seq aligner. *Bioinformatics* **29**, 15-21 (2013).
12. Y. Liao, G. K. Smyth, W. Shi, featureCounts: an efficient general purpose program for assigning sequence reads to genomic features. *Bioinformatics* **30**, 923-930 (2014).
13. C. Lee, E. Y. Kang, M. J. Gandal, E. Eskin, D. H. Geschwind, Profiling allele-specific gene expression in brains from individuals with autism spectrum disorder reveals preferential minor allele usage. *Nat Neurosci* **22**, 1521-1532 (2019).
14. K. D. Hansen, R. A. Irizarry, Z. Wu, Removing technical variability in RNA-seq data using conditional quantile normalization. *Biostatistics* **13**, 204-216 (2012).
